# LXRα/SCD1-Mediated Endoplasmic Reticulum-Mitochondria Crosstalk in Inhibiting Neuronal Ferroptosis after Spinal Cord Injury

**DOI:** 10.34133/research.1077

**Published:** 2026-02-10

**Authors:** Pan Jiang, Yiqian Luo, Daoqiang Huang, Jiale He, Hong Li, Longyou Xiao, Senyu Yao, Mao Pang, Limin Rong, Bin Liu

**Affiliations:** ^1^ Department of Spine Surgery, The Third Affiliated Hospital of Sun Yat-Sen University, Guangzhou 510630, China.; ^2^ Spinal Surgery Department, Beijing Jishuitan Hospital Guizhou Hospital, Guiyang 550014, China.; ^3^ Guangdong Provincial Center for Engineering and Technology Research of Minimally Invasive Spine Surgery, Guangzhou 510630, China.; ^4^ Guangdong Provincial Center for Quality Control of Minimally Invasive Spine Surgery, Guangzhou 510630, China.

## Abstract

Spinal cord injury (SCI) causes extensive neuronal loss, in which ferroptosis is critically involved. Although lipid transport at endoplasmic reticulum-mitochondria contact sites (ERMCSs) has been implicated in facilitating ferroptosis, the neuron-specific regulatory mechanisms remain elusive. Here, we show that neuronal ferroptosis is characterized by excessive ERMCS formation. Mechanistically, a systematic screening revealed the down-regulation of stearoyl-CoA desaturase 1 (SCD1), a critical enzyme in the synthesis of monounsaturated fatty acids (MUFAs) in neurons, following SCI (in vivo) or erastin treatment (in vitro). We demonstrated that SCD1 deficiency is the driving force behind aberrant ERMCS expansion, leading to increased lipid peroxidation and neuronal ferroptosis. Conversely, SCD1 overexpression could reverse these effects. Furthermore, we identified liver X receptor alpha (LXRα) as a direct transcriptional activator of SCD1. Pharmacological activation of LXRα with T0901317 upregulated SCD1 expression, which in turn restrained ERMCS formation, elevated MUFA levels, and ultimately inhibited neuronal ferroptosis. In murine SCI models, both adeno-associated virus-mediated neuronal SCD1 overexpression and LXRα agonist treatment effectively mitigated excessive ERMCS, reduced lesion size, preserved neuronal architecture, and improved functional recovery. Collectively, our study establishes the LXRα–SCD1 axis as a novel and druggable pathway for reducing neuronal loss and improving functional recovery by modulating ERMCS-dependent lipid exchange dynamics, revealing promising therapeutic targets after central nervous system trauma.

## Introduction

Spinal cord injury (SCI) is a severe neurological disorder that leads to massive damage to spinal cord tissue [1]. Each year, approximately 700,000 new cases are reported worldwide, mostly resulting from traumatic incidents such as traffic accidents [2]. Currently, there are no safe and effective repair strategies available, making the resulting functional impairments and growing socioeconomic burden a global health challenge [3]. This underscores the urgent need to elucidate the molecular mechanisms underlying SCI and to identify potential therapeutic targets.

In recent years, ferroptosis, a form of regulated cell death driven by iron dependency and lipid peroxidation, has become a central focus of SCI research [4]. During the early stage of SCI, the intricate injury microenvironment triggers a series of pathological events, such as inflammatory activation, lipid peroxidation, and an overproduction of reactive oxygen species (ROS), ultimately resulting in neuronal and glial cell death via necrosis or programmed pathways [5]. In particular, vascular disruption and erythrocyte lysis after SCI result in iron overload, thereby providing a permissive environment for ferroptosis. Growing evidence indicates that ferroptosis plays a crucial role in the early neuronal loss following SCI [6–8].

Recent studies by Sassano et al. [9] demonstrated that endoplasmic reticulum-mitochondria contact sites (ERMCSs) act as essential platforms for the initiation of phospholipid peroxide formation, which subsequently diffuse to mitochondria, causing oxidative injury and triggering ferroptosis. These organelles communicate through ERMCSs, which mediate the exchange of metabolites and signals. While the endoplasmic reticulum (ER) is the principal site of lipid synthesis and processing, mitochondria are the primary hubs of energy metabolism and ROS generation. Following SCI, excessive production of ROS and pronounced mitochondrial dysfunction have been observed, accompanied by an abnormal increase in ERMCSs [10,11]. However, whether these pathophysiological factors act synergistically to exacerbate secondary neuronal damage after SCI remains unclear.

Lipid metabolism dysfunction is an important contributor to ferroptosis in SCI. It is noteworthy that neurons contain a high proportion of polyunsaturated fatty acids (PUFAs), a feature that contributes to their heightened susceptibility to membrane lipid peroxidation and ferroptosis [12–15]. For instance, the upregulation of acyl-CoA synthetase long-chain family member 4 (ACSL4) facilitates PUFA enrichment, markedly increasing ferroptosis sensitivity [16]. In contrast, monounsaturated fatty acids (MUFAs) exhibit stronger antioxidative properties and confer resistance against oxidative stress [17]. These results underscore the crucial role of maintaining PUFA–MUFA homeostasis in regulating neuronal ferroptosis susceptibility.

Among lipid metabolic regulators, stearoyl-CoA desaturase 1 (SCD1) is a multifunctional enzyme family with distinct isoforms that exert diverse roles across different pathological contexts. Of particular significance, SCD1 is an essential ER-localized enzyme that catalyzes the conversion of saturated fatty acids into MUFAs [18,19]. As the rate-limiting enzyme for MUFA biosynthesis, SCD1 mitigates lipid peroxidation by elevating MUFA levels, and its overexpression has been shown to substantially enhance cellular resistance to ferroptosis [20,21]. Experimental evidence indicates that suppression of SCD1 expression strengthens ERMCSs, promotes ERMCS formation, and aggravates mitochondrial lipid radical accumulation and dysfunction [22,23]. Nevertheless, the precise role of SCD1 in SCI pathophysiology, particularly in coordinating lipid metabolism between the ER and mitochondria in neurons, remains poorly understood. Furthermore, the upstream regulatory mechanisms controlling SCD1 expression and activity have yet to be fully elucidated.

This study demonstrates that SCI results in the down-regulation of SCD1, a crucial enzyme involved in MUFA synthesis. This reduction drives the aberrant expansion of ERMCSs, which leads to enhanced lipid peroxidation and neuronal ferroptosis. Mechanistically, the transcription factor liver X receptor alpha (LXRα) directly activates SCD1 transcription. This activation enhances cellular antioxidant capacity by enriching MUFAs within membrane phospholipids. Moreover, by coordinating lipid exchange between mitochondria and the ER, LXRα–SCD1 signaling promotes mitochondrial lipid renewal and optimizes the lipid composition at ERMCSs, thereby enhancing mitochondrial resilience to ROS. Collectively, our study establishes the LXRα–SCD1 axis as a novel and druggable pathway for reducing neuronal loss and improving functional recovery by modulating ERMCS-dependent lipid exchange dynamics, revealing promising therapeutic targets after central nervous system trauma.

## Results

### SCI induces aberrant ERMCS expansion, ER stress, mitochondrial oxidative stress, and ferroptosis

**Fig. 1.**
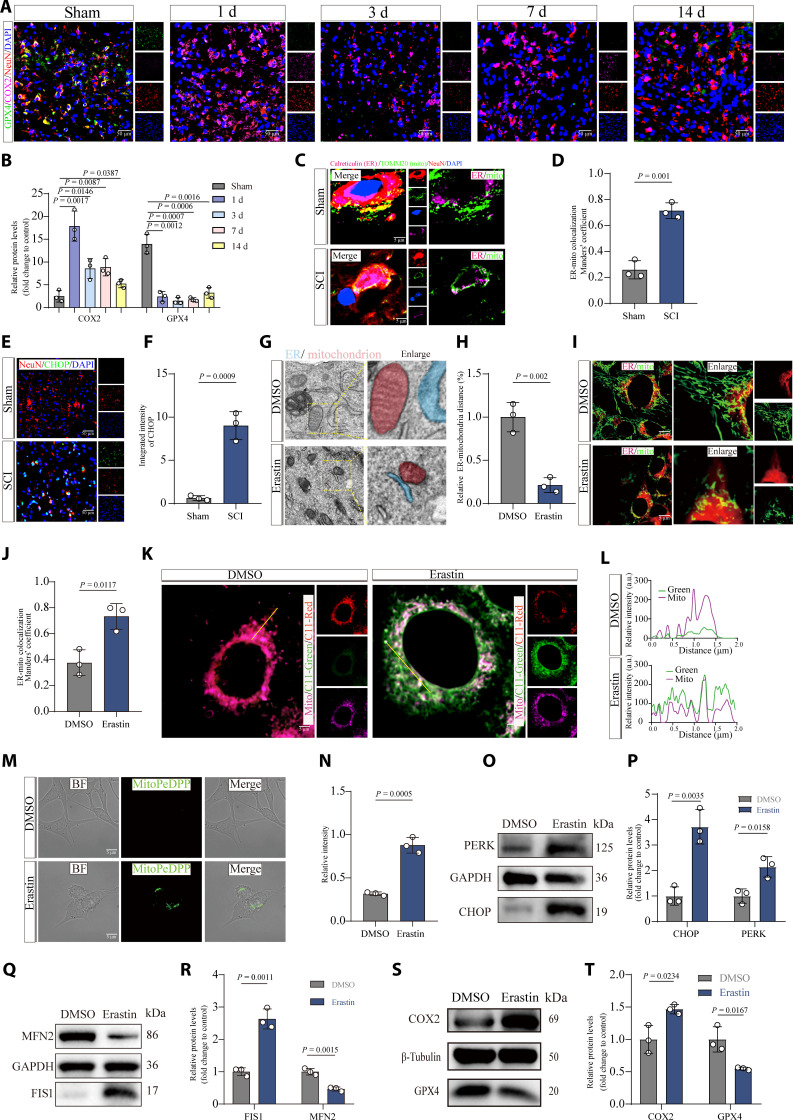
Enhanced endoplasmic reticulum (ER)-mitochondria contact is associated with oxidative stress and ferroptosis after spinal cord injury (SCI). (A and B) Immunofluorescence of COX2 and GPX4 expression at indicated time points post-SCI (7 d post-SCI). (C and D) Confocal microscopy showing increased ER-mitochondria contacts in neurons. (E and F) CHOP immunofluorescence in spinal cord neurons (7 d post-SCI). (G and H) Transmission electron microscopy (TEM) images quantifying mitochondrial ultrastructure and ER-mitochondria distance. (I and J) ER-Tracker/MitoTracker co-staining in HT22 cells after erastin (1 μM, 24 h). (K to N) C11-BODIPY and MitoPeDPP staining showing elevated mitochondrial lipid peroxidation. (O to T) Western blot analysis of PERK, CHOP, FIS1, MFN2, COX2, and GPX4 with densitometric quantification. Data are presented as mean ± SD from 3 independent biological replicates per group. Statistical significance was set at *P* < 0.05; ns, not significant. BF, bright field; GAPDH, glyceraldehyde-3-phosphate dehydrogenase.

In a mouse SCI model, immunofluorescence analysis revealed persistent dysregulation of ferroptosis-associated markers. COX2 expression was markedly increased, while GPX4 was significantly reduced at the lesion epicenter (Fig. 1A and B), consistent with enhanced oxidative stress. Previous studies have suggested that excessive contact between mitochondria and the ER is a critical trigger of lipid peroxidation [9]. In line with this, we observed that oxidative stress after SCI was accompanied by a pronounced increase in ER-mitochondria contacts within neurons (Fig. 1C and D). Immunofluorescence of SCI spinal cords also showed a marked increase in CHOP-positive neurons in the peri-lesion region, confirming sustained ER stress in vivo (Fig. 1E and F). Similar changes were reproduced in vitro. HT22 cells exposed to erastin (1 μM, 24 h) exhibited enhanced ER-mitochondria colocalization, which was corroborated by transmission electron microscopy showing abnormal mitochondrial morphology and a shortened ER-mitochondria distance (Fig. 1G and H). Consistently, mitochondrial lipid peroxidation was elevated, as confirmed by C11-BODIPY/MitoTracker co-staining and MitoPeDPP fluorescence (Fig. 1I to N). At the molecular level, western blot analysis demonstrated robust activation of ER stress, indicated by upregulation of PERK and CHOP (Fig. 1O and P). Mitochondrial dynamics were also disrupted, with FIS1 upregulation and MFN2 down-regulation, reflecting enhanced mitochondrial fission (Fig. 1M and N). In parallel, increased COX2 and decreased GPX4 expression further supported the occurrence of ferroptosis (Fig. 1S and T). Together, these findings demonstrate that SCI induces persistent ER-mitochondria dysregulation, oxidative stress, and ferroptosis, providing mechanistic insight into neuronal vulnerability following injury.

### SCD1 overexpression alleviates erastin-induced excessive ERMCS formation and neuronal ferroptosis

**Fig. 2.**
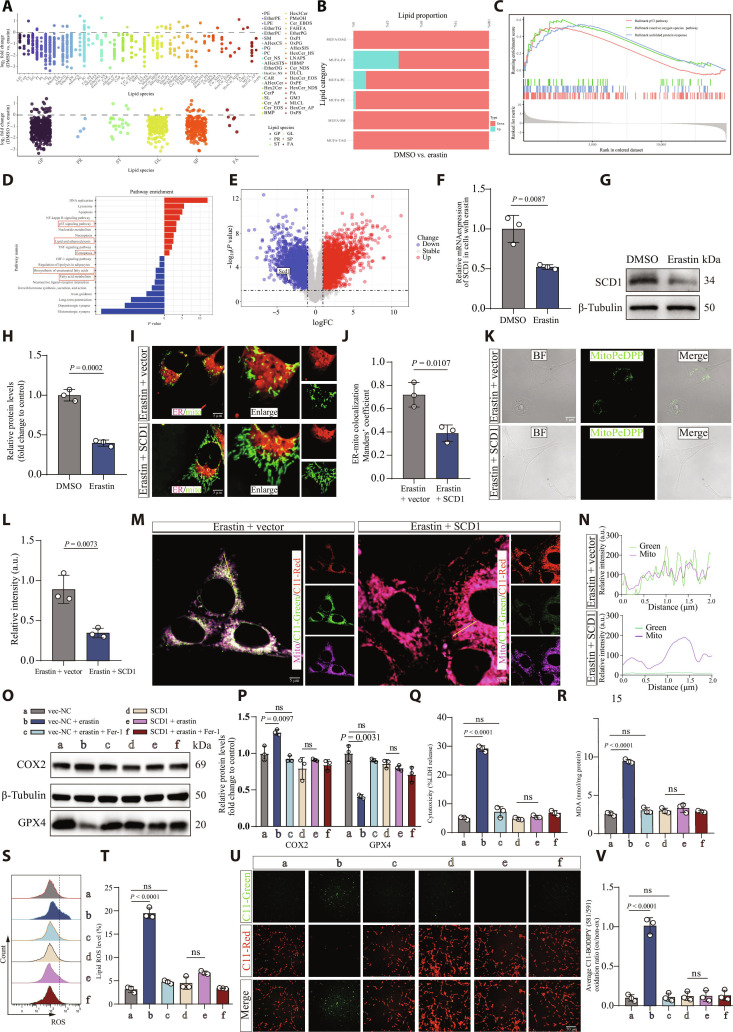
Stearoyl-CoA desaturase 1 (SCD1) protects neurons from erastin-induced ferroptosis by restoring lipid homeostasis and ER-mitochondria contacts. (A and B) Mitochondrial lipidomics showing decreased monounsaturated fatty acids (MUFAs) after erastin treatment. (C to E) Transcriptomic datasets (GSE45006) showing enrichment of reactive oxygen species (ROS), unfolded protein response (UPR), and p53 pathways and down-regulation of SCD1 in SCI. (F to H) Quantitative real-time polymerase chain reaction (qRT-PCR) and western blot confirming SCD1 suppression in erastin-treated HT22 cells. (I to N) Confocal imaging of ER-mitochondria contacts and lipid peroxidation (ER-Tracker/MitoTracker, MitoPeDPP, and C11-BODIPY), showing rescue by SCD1 overexpression. (O to V) Western blot, lactate dehydrogenase (LDH) release, malondialdehyde (MDA), ROS, and C11-BODIPY assays demonstrating enhanced ferroptosis with erastin, reversed by SCD1 overexpression or ferrostatin-1 (Fer-1). NC, negative control; vector, empty vector. Data are presented as mean ± SD from 3 independent biological replicates per group. Statistical significance was set at *P* < 0.05; ns, not significant. logFC, log fold change; mRNA, messenger RNA.

In HT22 cells, erastin treatment robustly reduced MUFAs and down-regulated cardiolipin and phosphatidylethanolamine, indicating impaired mitochondrial lipid homeostasis (Fig. 2A and B). Consistently, transcriptomic analysis of SCI datasets (GSE45006 and GSE166009) revealed marked suppression of fatty acid metabolism and unsaturated fatty acid synthesis, accompanied by the activation of oxidative stress pathways including ROS, unfolded protein response, and p53 signaling, together with reduced expression of SCD1 (Fig. 2C to E and Fig. S1A and B).

In vitro experiments verified that erastin inhibited both SCD1 transcription and protein expression in HT22 cells (Fig. 2F to H). Functional rescue assays demonstrated that SCD1 overexpression alleviated excessive ER-mitochondria contacts and reduced mitochondrial lipid peroxidation (Fig. 2I and J and Fig. S1C to G). Moreover, SCD1 overexpression reversed the erastin-induced markers of ferroptosis and functional abnormalities, including increased LDH release, elevated malondialdehyde (MDA) and ROS levels, mitochondrial depolarization (JC-1), and mitochondrial ROS accumulation (MitoSOX) (Fig. 2O to V and Fig. S1H to K). Notably, treatment with the ferroptosis inhibitor ferrostatin-1 (Fer-1) produced comparable protective effects.

Together, these findings identify SCD1 down-regulation as a central link between lipid metabolic imbalance and ferroptosis. Conversely, restoring SCD1 expression effectively preserved mitochondrial function and alleviated neuronal ferroptosis.

### Loss of SCD1 exacerbates ferroptosis, ER stress, and mitochondrial dysfunction in neurons

**Fig. 3.**
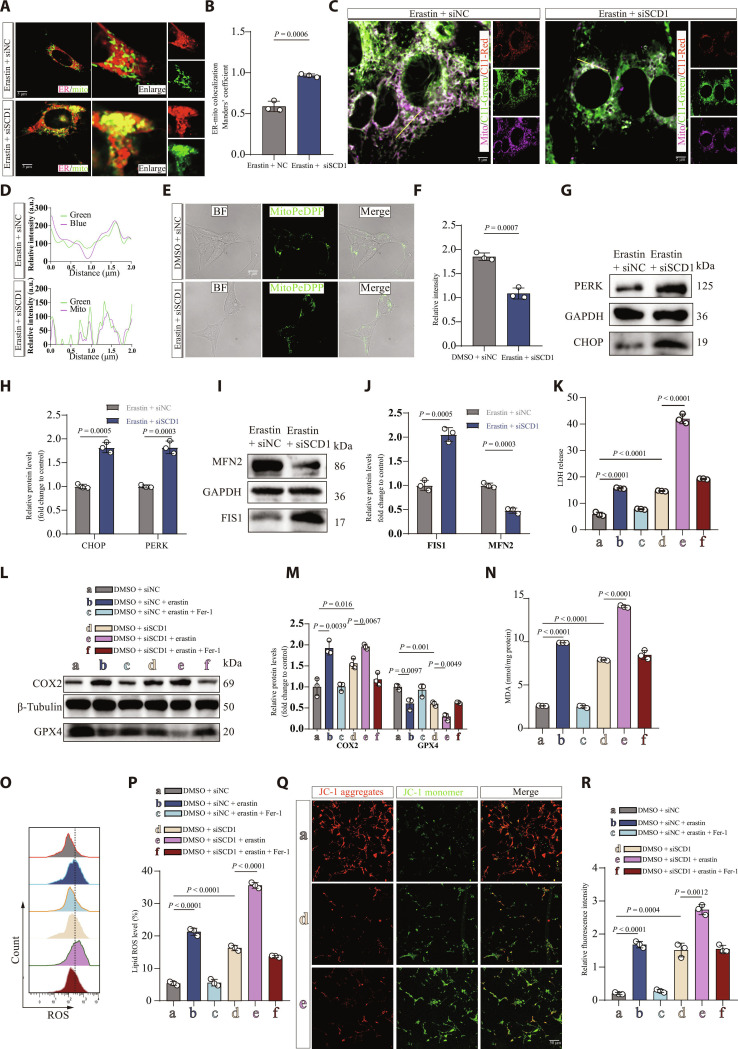
SCD1 deficiency aggravates ER-mitochondria coupling and ferroptosis. (A to F) Confocal imaging showing increased ER-mitochondria contacts and lipid peroxidation in SCD1-knockdown cells. (G to J) Western blot revealing elevated ER stress markers (PERK and CHOP) and disrupted mitochondrial dynamics (MFN2 and FIS1). (K to N) Functional assays (LDH, MDA, and COX2/GPX4) showing exacerbated ferroptosis, partially rescued by Fer-1. (O to R) ROS detection (DCFH-DA) detection and JC-1 staining confirming oxidative stress and mitochondrial depolarization, reversed by Fer-1. Data are presented as mean ± SD from 3 independent biological replicates per group. Statistical significance was set at *P* < 0.05; ns, not significant.

In HT22 cells, siRNA-mediated knockdown of SCD1 (Fig. S2A and B) markedly increased ER-mitochondria interactions (Fig. 3A and B), which was accompanied by elevated mitochondrial lipid peroxidation, as demonstrated by C11-BODIPY and MitoPeDPP assays (Fig. 3C and D). Molecularly, SCD1 deficiency intensified ER stress, with PERK and CHOP expression significantly upregulated (Fig. 3G and H). Mitochondrial dynamics were also disrupted, as indicated by decreased MFN2 and increased FIS1, suggesting enhanced mitochondrial fission (Fig. 3I and J). Functionally, SCD1 depletion aggravated erastin-induced injury. This was reflected by elevated LDH release and MDA accumulation, increased ROS production, loss of mitochondrial membrane potential (JC-1), and enhanced oxidative signals detected by MitoSOX and C11-BODIPY staining (Fig. 3K and N to R and Fig. S2D to H). Western blot analysis further confirmed ferroptotic progression, with COX2 markedly upregulated and GPX4 down-regulated (Fig. 3L and M). Importantly, treatment with Fer-1 (5 μM) partially reversed these pathological changes, mitigating oxidative stress, restoring mitochondrial function, and reducing ferroptosis. Collectively, these findings demonstrate that SCD1 deficiency considerably exacerbates oxidative injury, mitochondrial dysfunction, and ferroptosis, highlighting its essential protective role in maintaining neuronal homeostasis.

### Transcriptional regulation of SCD1 by an LXRα agonist maintains lipid homeostasis and protects against ferroptosis in neurons

**Fig. 4.**
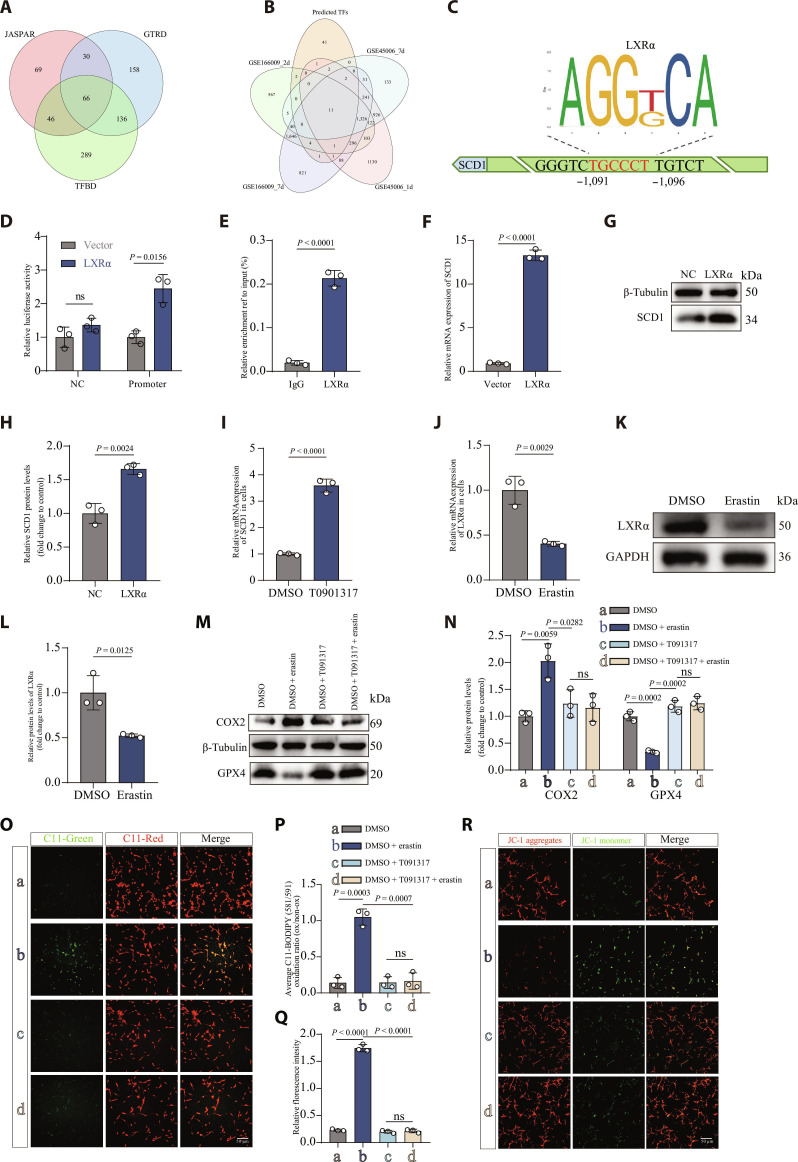
Liver X receptor alpha (LXRα) transcriptionally activates SCD1 and modulates ferroptosis susceptibility. (A to C) Bioinformatic prediction identifying LXRα as a potential SCD1 regulator (transcription factor [TF] overlap, JASPAR motif). (D and E) Dual-luciferase reporter and ChIP-qPCR confirming LXRα binding to the SCD1 promoter. (F to H) qRT-PCR and western blot showing increased SCD1 expression after LXRα overexpression. (I to L) Pharmacological data showing SCD1 induction by T0901317 and LXRα down-regulation by erastin. (M to R) Functional assays showing that T0901317 attenuated ferroptosis (COX2/GPX4, lipid peroxidation, and JC-1). Data are presented as mean ± SD from 3 independent biological replicates per group. Statistical significance was set at *P* < 0.05; ns, not significant.

Transcription factor prediction integrated with SCI transcriptomic datasets (GSE45006 and GSE166009) identified 11 candidate regulators of SCD1 (Fig. 4A and B and Tables S1 and S2). Among these, bioinformatics analysis suggested that LXRα could directly bind the SCD1 promoter (Fig. 4C). Quantitative real-time polymerase chain reaction (qRT-PCR) screening further demonstrated that most candidates were weakly expressed in neurons, whereas only LXRα showed a strong positive correlation with SCD1 expression (Fig. 4F and Fig. S3B to D). Assays validated this regulatory relationship. Overexpression of LXRα notably increased both SCD1 messenger RNA and protein levels (Fig. 4G and H). Dual-luciferase reporter assays and chromatin immunoprecipitation-quantitative polymerase chain reaction (ChIP-qPCR) further verified direct binding of LXRα to the SCD1 promoter (Fig. 4D and E and Fig. S3A). Consistently, pharmacological activation with the LXRα agonist T0901317 (500 nM) [24] induced robust SCD1 upregulation (Fig. 4I and Fig. S3E and F). In contrast, erastin treatment (1 μM, 24 h) suppressed LXRα transcription and protein expression (Fig. 4J to L).

Importantly, T0901317 treatment alleviated ferroptosis by normalizing the erastin-induced increase in COX2 and decrease in GPX4 (Fig. 4M and N). It also substantially improved multiple functional parameters, including reduced LDH release, MDA accumulation, total and mitochondrial ROS (MitoSOX), lipid peroxidation (C11-BODIPY), and recovery of mitochondrial membrane potential (JC-1) (Fig. 4O to R and Fig. S3G to L). Moreover, we demonstrated that LXRα knockdown in erastin-induced HT22 cells exacerbated ferroptosis (Fig. S3M to P), as evidenced by increased lipid peroxidation and diminished mitochondrial membrane potential (Fig. S3Q to T). Notably, the LXRα agonist T0901317 reversed these effects, and this protection was abolished when LXRα was knocked down, further confirming that T0901317 functions through LXRα.

Collectively, these findings establish LXRα as a direct transcriptional activator of SCD1. Moreover, these demonstrate that the LXRα–SCD1 axis plays a pivotal protective role in maintaining lipid homeostasis and conferring resistance to ferroptosis.

### LXRα protects against neuronal ferroptosis by preserving ER-mitochondria homeostasis in an SCD1-dependent manner

**Fig. 5.**
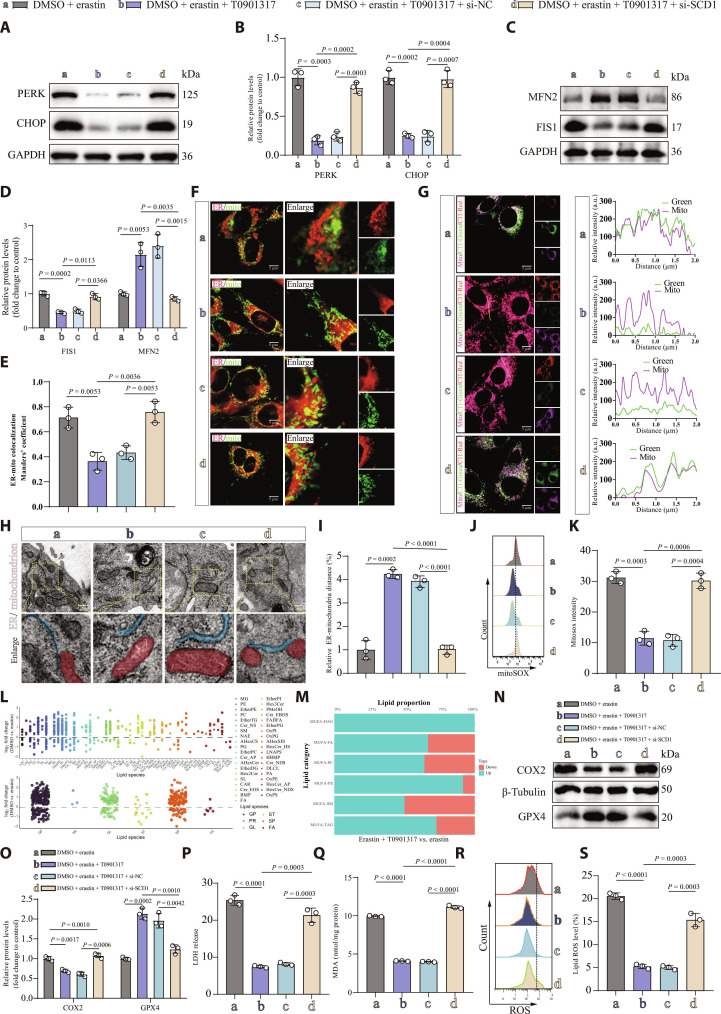
LXRα activation rescues erastin-induced ferroptosis through SCD1-dependent ER-mitochondria regulation. (A to D) Western blot showing that T0901317 reduced ER stress and restored mitochondrial dynamics, effects abolished by SCD1 knockdown. (E to G) Confocal imaging demonstrating normalization of ER-mitochondria contacts and lipid peroxidation with T0901317, dependent on SCD1. (H and I) TEM showing preserved mitochondrial ultrastructure and ER-mitochondria spacing. (J to M) MitoSOX and lipidomics showing reduced mitochondrial ROS, restored MUFAs/phospholipids, and reduced cardiolipin oxidation. (N and O) Ferroptosis markers were normalized by T0901317 in an SCD1-dependent manner. (P to S) Functional assays showing reduced cell death (LDH), MDA, and ROS, effects reversed by SCD1 knockdown. Data are presented as mean ± SD from 3 independent biological replicates per group. Statistical significance was set at *P* < 0.05; ns, not significant.

In HT22 cells, treatment with the LXRα agonist T0901317 markedly alleviated erastin-induced stress responses. Specifically, T0901317 reduced ER stress by suppressing PERK and CHOP upregulation (Fig. 5A and B), corrected mitochondrial dynamics by restoring MFN2 and lowering FIS1 expression (Fig. 5C and D), and diminished excessive ER-mitochondria contacts and lipid peroxidation (Fig. 5E to G and Fig. S4A and B). Ultrastructural analysis further confirmed improvements in mitochondrial morphology and normalization of ER-mitochondria proximity (Fig. 5H and I).

Lipidomic profiling demonstrated that T0901317 restored mitochondrial lipid homeostasis, reversing the erastin-induced depletion of MUFAs and key phospholipids (Fig. 5L and M). Functionally, T0901317 reduced mitochondrial ROS accumulation (Fig. 5J and K), normalized ferroptosis markers by lowering COX2 and restoring GPX4 expression (Fig. 5N and O), and improved cellular outcomes including reduced LDH release, decreased MDA and ROS levels, and recovery of mitochondrial membrane potential (JC-1) (Fig. 5P to S and Fig. S4E).

Importantly, all of these protective effects were abolished by SCD1 knockdown. In the absence of SCD1, T0901317 failed to enhance SCD1 expression or activity (Fig. S4G and H), and its protective capacity against ER stress, mitochondrial dysfunction, and ferroptosis was lost.

Together, these findings demonstrate that the neuroprotective effects of T0901317 are mediated by SCD1. By preserving ER-mitochondria homeostasis, restoring lipid metabolism, and suppressing ferroptotic signaling, the LXRα–SCD1 axis emerges as a critical regulator of neuronal resilience under oxidative stress.

### The LXRα–SCD1 axis attenuates ERMCS and protects against neuronal ferroptosis after SCI

**Fig. 6.**
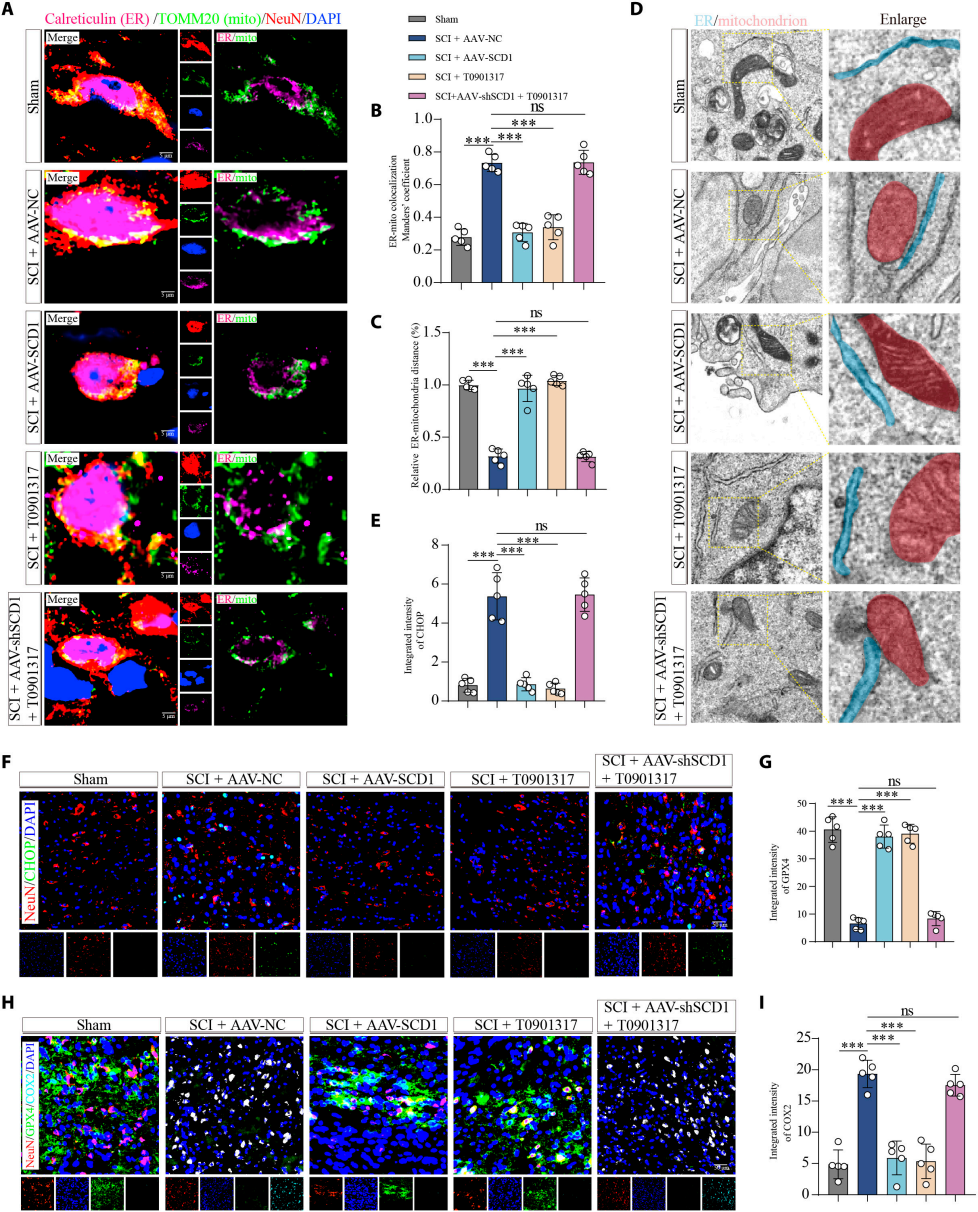
Neuron-specific SCD1 overexpression and LXRα activation preserve ER-mitochondria homeostasis after SCI. (A and B) Triple immunofluorescence (NeuN, calreticulin, and TOMM20) showing restored ER-mitochondria contacts post-SCI, enhanced by AAV-SCD1 or T0901317, abolished by SCD1 knockdown. AAV, adeno-associated virus. (C and D) TEM quantification confirming improved ER-mitochondria spacing, crista integrity, and ER continuity. (E and F) CHOP immunofluorescence showing reduced ER stress in NeuN+ neurons. (G to I) immunofluorescence showing the normalization of the neuronal COX2/GPX4 ratio in peri-lesion neurons following SCD1 overexpression or T0901317. Data are mean ± SD (*n* = 5 mice/group). *P < 0.05; **P < 0.01; ***P < 0.001; ns, not significant.

In vivo studies demonstrated that both neuron-specific SCD1 overexpression and pharmacological activation of LXRα with T0901317 markedly alleviated SCI-induced ER-mitochondria dysfunction. Immunofluorescence analysis showed that the injury-associated increase in ER-mitochondria colocalization and ER stress was markedly reduced following either intervention. Consistently, electron microscopy confirmed that both treatments improved mitochondrial morphology and restored ER-mitochondria spacing, reversing the shortened distance observed after SCI (Fig. 6A to D and Fig. S5A and B).

Molecularly, these interventions suppressed neuronal ER stress, reducing CHOP upregulation (Fig. 6E and F and Fig. S5C) while also normalizing ferroptosis markers by decreasing COX2 expression and restoring GPX4 levels. However, when SCD1 was silenced, the protective effects of T0901317 were largely abolished, with ER-mitochondria contacts and COX2/GPX4 ratios remaining comparable to those of untreated SCI (Fig. 6G to I and Fig. S5D).

Together, these results indicate that the neuroprotective effects of T0901317 are critically dependent on SCD1. By preserving ER-mitochondria homeostasis, reducing oxidative stress, and limiting ferroptotic signaling, the LXRα–SCD1 axis safeguards neuronal integrity following SCI.

### The LXRα–SCD1 axis promotes neuroprotection and functional locomotor recovery after SCI

**Fig. 7.**
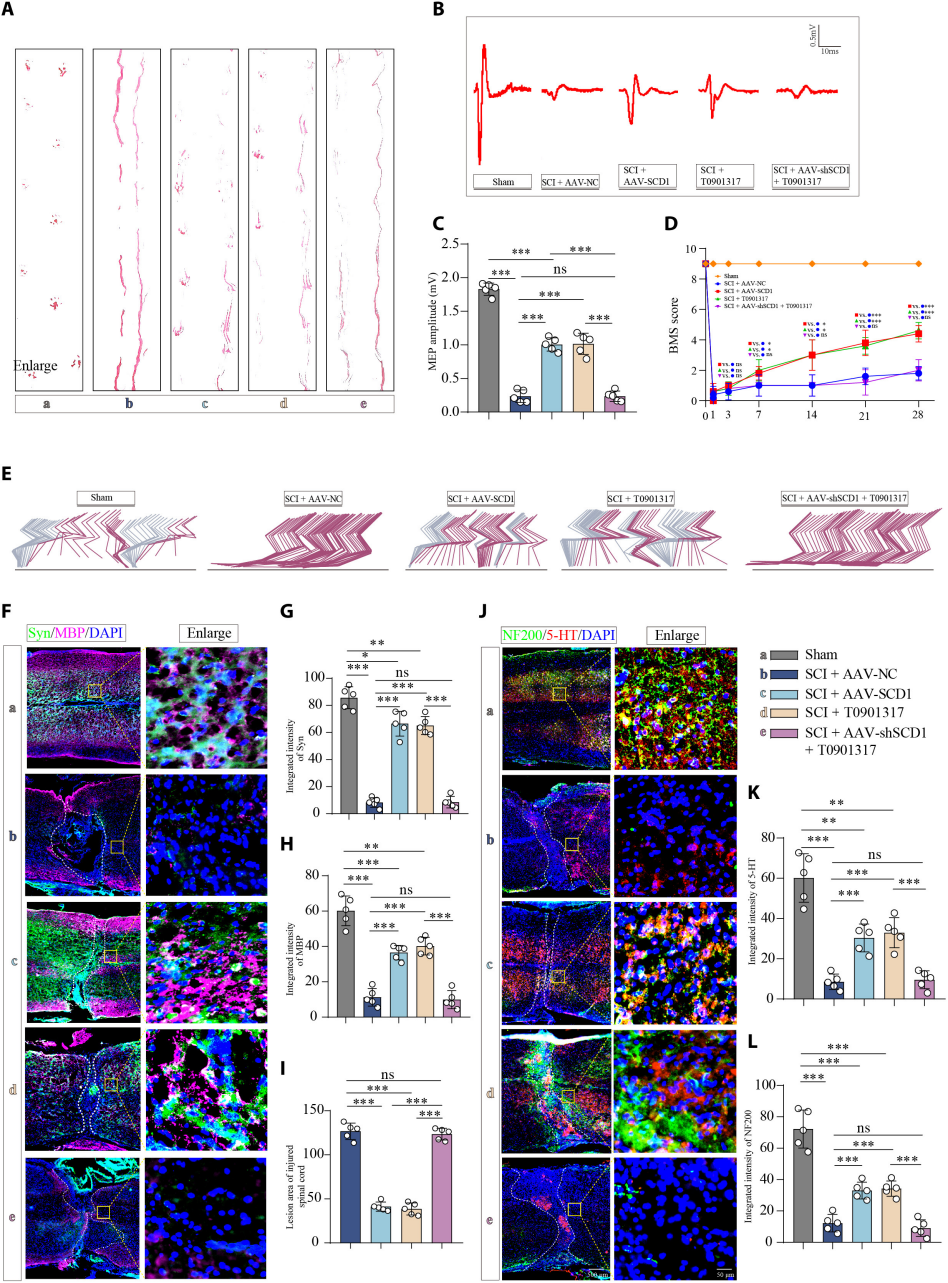
SCD1 overexpression and LXRα activation promote functional recovery after SCI. (A) Hind limb footprint analysis showing improved stride patterns with SCD1 overexpression or T0901317, abolished by SCD1 knockdown. (B and C) Electrophysiological recordings demonstrating the recovery of compound muscle action potentials. (D and E) Basso Mouse Scale (BMS) scores and gait kinematics confirming locomotor improvement. (F to I) Immunofluorescence showing increased synaptic density (Syn) and remyelination (myelin basic protein [MBP]) in the lesion epicenter. (J to L) Axonal preservation evidenced by 5-hydroxytryptamine-positive (5-HT+) fibers, NF200+ axons, and reduced lesion size. Data are mean ± SD (*n* = 5 mice/group). *P < 0.05; **P < 0.01; ***P < 0.001; ns, not significant. MEP, motor evoked potential.

In SCI mice, both neuron-specific SCD1 overexpression and pharmacological activation of LXRα with T0901317 significantly enhanced motor recovery at 4 weeks post-injury. Improvements were evident across multiple functional measures, including hind limb footprints, gait trajectories, Basso Mouse Scale scores, and sciatic-nerve-evoked potentials (Fig. 7A to E and Fig. S6A to E). In contrast, SCD1 knockdown markedly attenuated the beneficial effects of T0901317. Verification assays confirmed that neuronal SCD1 expression was robustly increased following overexpression (Fig. S6F and G).

Histological analyses revealed that both SCD1 overexpression and T0901317 treatment restored the expression of synaptic (Syn) and myelin (MBP) markers and substantially reduced lesion size (Fig. 7F to I and Fig. S6H to K). Moreover, serotonergic fibers (5-HT) and axonal markers (NF200) were preserved, indicating improved axon and myelin integrity (Fig. 7J to L and Fig. S6L to N). These protective effects, however, were abolished in the context of SCD1 knockdown, further underscoring the necessity of SCD1 for LXRα-mediated neuroprotection.

Taken together, these results demonstrate that the neuroprotective effect of T0901317 depends on SCD1. By maintaining neuronal structural integrity and promoting axonal and myelin repair, the LXRα–SCD1 axis plays a pivotal role in driving the recovery of function following SCI.

## Discussion

**Fig. 8.**
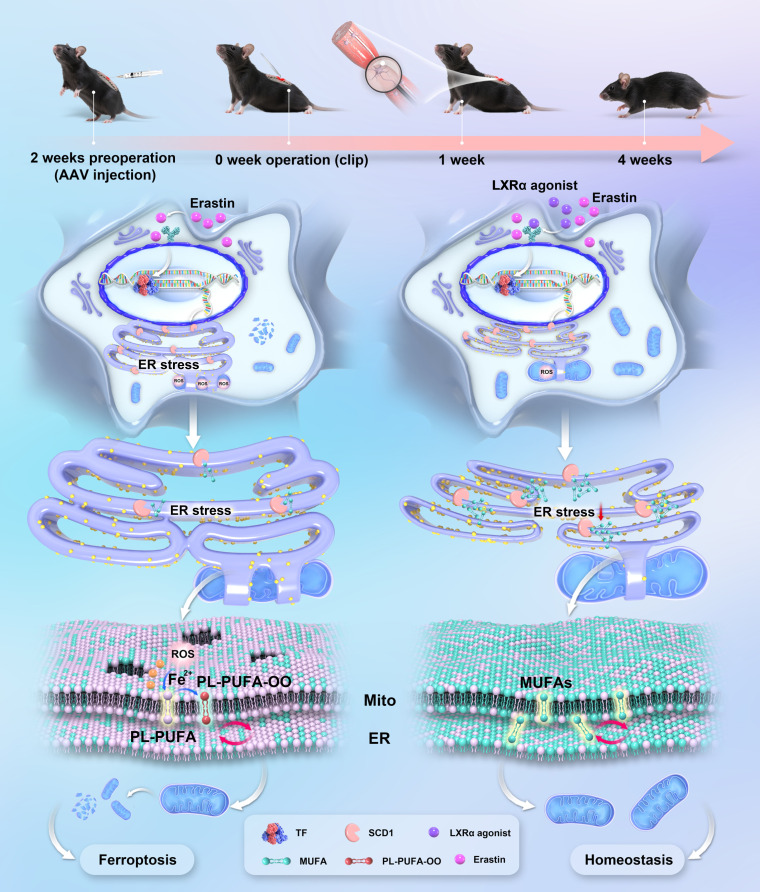
Illustration of the mechanism of LXRα or SCD1 and its proposed therapeutic mechanism in SCI treatment.

SCI is characterized by excessive mitochondrial oxidative stress, lipid metabolic dysregulation [25], and neuronal ER stress [26]. Ferroptosis is increasingly acknowledged as a key contributor to neuronal death following SCI [27]. The increased ERMCSs and enhanced lipid peroxidation drive ferroptosis [9]. The balance between MUFAs and PUFAs is particularly critical for cellular responses to oxidative stress [28]. Herein, we demonstrated that LXRα activation transcriptionally upregulates SCD1, thereby promoting MUFA synthesis (Fig. 8). This remodeling of mitochondrial lipid composition enhances antioxidant capacity and reduces ERMCSs, ultimately attenuating ferroptosis in neurons. SCD1, an ER-localized enzyme, serves as a key regulator of lipid metabolism by synthesizing MUFAs [29]. Transcriptomic analysis of spinal cord tissue revealed a pronounced down-regulation of SCD1 following SCI, which may exacerbate neuronal loss. Functional studies showed that SCD1 overexpression, together with the activation of the transcription factor LXRα, enhanced MUFA synthesis, increased mitochondrial MUFAs, suppressed mitochondrial lipid peroxidation, and decreased ERMCSs. Furthermore, the pronounced erastin-induced alterations in neuronal ferroptosis markers observed in vitro were successfully recapitulated in our in vivo models, confirming the consistency of ferroptotic responses across experimental systems and reinforcing our previous findings [30]. In vivo, this intervention promoted neuronal survival after SCI, thereby providing a histological basis for neural repair. These findings establish SCD1 as a critical neuroprotective factor in maintaining neuronal homeostasis.

ERMCSs are essential structural platforms that mediate communication between the ER and mitochondria [31,32]. Increasing evidence implicates ERMCSs in the pathogenesis of diverse diseases. Physiologically, these contacts provide a structural basis for lipid exchange, calcium transfer, and other forms of interorganelle communication [33]. Under pathological stress, however, ERMCSs exhibit a dual function: they facilitate mitochondrial support in alleviating ER stress through direct physical interaction while also enabling the transfer of peroxidized lipids from mitochondria to the ER for clearance [22,33]. Excessive contact under conditions of elevated oxidative stress, in turn, amplifies phospholipid peroxidation and contributes to the initiation and progression of ferroptosis [9]. In our study, oxidative stress enhanced ER-mitochondria contacts, increased mitochondrial lipid peroxidation, altered ferroptosis marker expression, and reduced mitochondrial MUFA levels. Conversely, overexpression of LXRα/SCD1 alleviated mitochondrial lipid peroxidation and restored MUFA proportions in mitochondrial membranes and ERMCS formation. These findings suggest that MUFAs synthesized by SCD1 may be transported to mitochondria via ERMCS lipid transfer, where they enhance antioxidant capacity and mitigate lipid peroxidation. This provides novel mechanistic insights into SCD1’s neuroprotective role in SCI.

Despite progress, the regulatory network linking ferroptosis and lipid peroxidation in neurons remains incompletely understood. Using differential gene expression analysis and JASPAR-based transcription factor prediction, we identified potential binding sites for LXRα at the SCD1 promoter. Mechanistic studies further confirmed the importance of the LXRα–SCD1 axis in SCI. Both LXRα and SCD1 showed parallel down-regulation under neuronal oxidative stress. While earlier studies suggested an antioxidant role for LXRα [34], its contribution to ferroptosis had not been clarified. Our results demonstrate that LXRα specifically binds to and activates the SCD1 promoter, thereby transcriptionally upregulating SCD1 to reinforce neuronal antioxidant defenses. Pharmacological activation of LXRα markedly increased SCD1 expression, improved neuronal survival, and facilitated motor function recovery in vivo. Conversely, these neuroprotective effects were notably diminished in SCD1-knockdown mice, indicating that LXRα-mediated protection is dependent on SCD1. Thus, SCD1 emerges as the central molecular hub mediating LXRα’s neuroprotective effects.

Although SCD1’s involvement in ferroptosis has been reported [35–37], its precise MUFA-mediated antioxidant mechanisms remain to be fully elucidated. Here, we demonstrate that LXRα transcriptionally regulates SCD1 expression. Functionally, SCD1 simultaneously alleviates neuronal ER stress and modulates mitochondrial lipid composition through interorganelle lipid exchange, thereby enriching MUFAs at ERMCSs to prevent lipid peroxidation initiation. This dual regulation suppresses ferroptosis both in vitro and in vivo. Analogous to the SREBP1–SCD1 axis, which regulates organelle contacts via lipid composition [23], the LXRα–SCD1 axis reduces ER-mitochondria contacts, highlighting SCD1 as a core lipid metabolic regulator that confers resistance to ferroptosis by modifying organelle membrane composition. These results are consistent with the well-established lipid metabolic functions of LXRα [38], thereby reinforcing the importance of the LXRα–SCD1 axis in ferroptosis suppression.

Clinically, functional recovery after SCI remains a formidable challenge, with neuronal survival serving as the foundation for neural reconstruction. Currently, effective neuroprotective drugs for the acute phase of SCI are lacking. Our study highlights the central role of the LXRα–SCD1 axis in regulating lipid metabolism, mitigating oxidative stress, and suppressing ferroptosis. LXRα agonists demonstrate pleiotropic benefits, including the suppression of inflammatory gene expression, promotion of cholesterol efflux from macrophages, and protective effects against cardiovascular diseases such as atherosclerosis [39,40]. These agonists can also cross the blood–brain barrier, suggesting therapeutic potential in neurodegenerative contexts. In SCI, where robust neuroinflammation occurs, their established anti-inflammatory properties—combined with the antioxidant and antiferroptotic neuroprotection identified in this study—create a multitargeted therapeutic profile that enhances their translational promise. Although chronic use of LXRα agonists is associated with adverse effects such as elevated plasma triglycerides and hepatic lipid deposition [41], their application in the acute phase of SCI naturally mitigates this limitation by avoiding long-term exposure. Furthermore, future drug development efforts could focus on designing improved agonists with better safety profiles, thereby further expanding their clinical applicability.

This study has certain limitations. The inability to track specific lipid species, including individual MUFAs, remains an obstacle [42,43]. Consequently, the spatial dynamics of these lipids, despite their well-documented exchange at ERMCSs, cannot be directly traced. The transfer of ER-synthesized MUFAs to mitochondria was inferred indirectly from lipidomic profiling rather than directly demonstrated, and further verification would be possible in the future when conditions are favorable with advancements in technology. The mechanistic analyses relied primarily on cell and mouse models, which may not fully recapitulate human SCI pathology. Future studies employing real-time imaging, tracer-based assays, and clinically relevant models are needed to validate and extend these findings.

To conclude, this study provides a comprehensive demonstration of the neuroprotective role of SCD1 in SCI and identifies the LXRα–SCD1 axis as a critical regulatory pathway that modulates lipid metabolism and ER-mitochondria interactions. By alleviating oxidative stress and ferroptosis, this axis contributes to improved functional recovery. Collectively, these findings not only enhance our comprehension of SCI pathogenesis but also reveal novel therapeutic targets for neuroprotection and repair.

## Materials and Methods

### SCI model and animal adeno-associated virus-9 injection and processing

All animal procedures were conducted following protocols authorized by the Ethical Review Board of Sun Yat-Sen University (SYSU-IACUC-2025-001375), China. Female C57BL/6J mice, aged 6 weeks, were acquired from Jiangsu GemPharma Tech and kept at the Animal Center of Sun Yat-Sen University under regulated environmental settings, maintaining a consistent temperature of 25 °C, a 12-h light/dark cycle and a relative humidity of 70%.

SCI was induced at the T10 level by compressing for 10 s. Briefly, we anesthetized the mice with sodium pentobarbital and then made a 2-cm incision along the dorsal thoracic spine. We performed a laminectomy at the T9 to T10 level to expose the spinal cord, which we clamped for 10 s, causing immediate hemorrhage at the injury site. After the injury, we sutured the muscle layers and closed the skin with wound clips. Postoperative care included recovery on a warming blanket, analgesia using buprenorphine, and cyclosporin. A administration to prevent infection. We performed manual bladder expression daily until the mice resumed spontaneous urination.

Two microliters (2 μl) of adeno-associated virus-9-SCD1, shSCD1, or control vector (OBIO, China; titer: 1 × 10^13^ VG/ml) under the control of a neuron-specific synaptic protein promoter was stereotaxically microinjected into the T9 to T10 spinal cord segment using a 33-gauge Hamilton syringe at a rate of 5 μl/min. The injection needle was left in place for an additional 5 min postinjection to minimize backflow. Compression injury was applied at the injection site. Successful overexpression of SCD1 was confirmed by immunofluorescence. The administration of T0901317 was performed according to previously established methods [44].

### Evaluation of the functional recovery of rats with SCI

We assessed motor function recovery in mice using the Basso Mouse Scale scoring system. The mice were allowed to move freely for 5 min within a 90-cm open field, during which hind limb movement and interlimb coordination were closely observed. Assessments were conducted on postoperative days 1, 3, 7, 14, 21, and 28. Evaluators were blinded to group assignments, and each assessment was performed 3 times, with scores recorded immediately. To further assess limb coordination, the hind paws were stained with red ink, and footprints were collected for gait analysis. Meanwhile, bipedal locomotion was recorded using a motion capture system. Then, reflective markers were placed bilaterally on key anatomical landmarks of the mice, including the malleolus (ankle), lateral condyle (knee), greater trochanter (hip), iliac crest, and the base of the metatarsophalangeal joint. The positions of these markers were then reconstructed in 2 dimensions to analyze hind limb movement.

### Histological analysis

Six weeks following SCI, the mice were heavily anesthetized using sodium pentobarbital and subsequently euthanized. Spinal cord tissues were fixed in 4% (w/v) paraformaldehyde for 24 h and then cryosectioned at a thickness of 14 μm using a cryostat. For paraffin embedding, tissues underwent a 48-h fixation, followed by a 4-h rinse in running water, graded ethanol dehydration, and overnight xylene clearing. Samples were paraffin-embedded, and 7-μm sections were prepared using a microtome. After immersing in water for 5 min, the sections were first stained with hematoxylin for 5 min, followed by eosin for 2 min (Solarbio). Cellular and extracellular matrix morphology was examined under a light microscope. Additionally, neurofilament protein immunostaining and other relevant markers were used to assess neuronal integrity and function.

### Motor evoked potential detection

Motor evoked potentials were assessed to determine the functional status of the lower limb motor neural pathway 6 weeks following SCI. Electrophysiological recordings were performed using the BL-420A/F Data Acquisition and Analysis System (TECHMAN SOFT). Responses were recorded from the sciatic nerve on the opposite side, focusing on the latency and amplitude of the initial evoked potential as primary indicators of motor neuron conduction and functional recovery.

### Culture cell line

HT22 cells, originally isolated from the murine hippocampus, were obtained from the American Type Culture Collection (Rockville, MD, USA). These cells were cultured in Dulbecco’s modified Eagle medium (DMEM; Gibco, New Zealand) supplemented with 10% fetal bovine serum (Gibco, New Zealand) and 1% penicillin–streptomycin (4 mg/ml; Sigma-Aldrich) at 37 °C in a humidified incubator with 5% CO_2_. Experimental procedures were carried out when cell confluency reached between 60% and 80%. Subsequently, the cells were subjected to 1 μM erastin-induced treatment (24 h) [45] or Fer-1 [46]or other experimental manipulations, including transfection.

### Transfection

For overexpression assays, cells were transfected with 1 μg of complementary DNA (cDNA) construct per well in a 6-well plate using Lipofectamine 3000 (Invitrogen, L3000015). For gene knockdown, cells received either a single or double transfection of siRNAs at concentrations ranging from 20 to 100 nM, also employing Lipofectamine 3000. In single-transfection protocols, cells were incubated with siRNA for 72 h or with cDNA constructs for 24 h. For double transfections, an initial dose of 50 nM siRNA (Sangon, China) was administered, followed by a second transfection with 50 nM siRNA after 24 to 48 h.

### Mitochondrial lipidomic and fatty acid profiling and analysis

Mitochondrial lipidomics was conducted to analyze lipid composition and oxidative alterations. Mitochondria were isolated from HT22 cells using a commercial extraction kit (Solarbio, China) and lipids were extracted by a modified Bligh–Dyer method. UHPLC–MS/MS analysis was carried out with a Vanquish UHPLC system connected to a Q-Exactive HF-X mass spectrometer (SCIEX, USA). Lipids were separated on a C18 reversed-phase column using a binary gradient, and data were acquired in both positive and negative ion modes with full MS and data-dependent MS/MS scans. Pooled QC samples were analyzed periodically. Differentially expressed lipids were identified based on variable importance in projection values greater than 1 and a *P* value of less than 0.05, followed by pathway enrichment analysis using the Kyoto Encyclopedia of Genes and Genomes and LIPID MAPS databases.

### Luciferase assay

Cells were co-transfected with the LXRα reporter vector and pRL control vector using Lipofectamine 3000 (Invitrogen, L3000015). After transfection, they were lysed with 1× Passive Lysis Buffer for 3 h, followed by centrifugation to collect the supernatant. Luciferase activity in the lysate was measured using the Dual-Luciferase Reporter Assay System (Promega, #E1980), with the sequential addition of LARII and Stop & Glo reagents, and luminescence was detected as per the manufacturer’s instructions.

### MDA measurement

Cellular MDA levels were measured using a commercial MDA assay kit (Solarbio, BC0025-100T/96S), based on the thiobarbituric acid reactive substances method. Following sample collection, the assay was carried out following the manufacturer’s protocol, and the MDA content was measured for each experimental group.

### Cell cytotoxicity assay

Cell cytotoxicity was measured using the LDH Assay Kit (Beyotime, C0017) following the manufacturer’s guidelines. Briefly, cells were cultured in 6-well plates and subjected to knockdown or overexpression treatments. After a 24-h incubation, the culture supernatant was collected. The supernatant was incubated with the LDH reaction mixture for 30 min, and the absorbance at 450 nm (with a reference wavelength of 650 nm) was recorded. Cytotoxicity was calculated as per the kit’s instructions, with cells treated with lysis buffer serving as the high (positive) control and untreated cells as the low (negative) control.

### Measurement of mitochondrial membrane potential

The mitochondrial membrane potential (Δψm), a key indicator of mitochondrial function, was assessed with the JC-1 probe (Servicebio, G1515). HT22 cells were collected, counted, and resuspended in PBS at a concentration of 1 × 10^5^ cells/ml. The JC-1 probe was diluted 1:500 in serum-free medium, following the manufacturer’s guidelines. Cells were incubated with the probe at 37 °C for 20 min, with gentle mixing every 3 to 5 min to ensure uniform dye distribution. After incubation, the cells were rinsed 3 times with serum-free medium to eliminate excess dye. A fluorescence microscope was then used for fluorescence analysis, following the provided protocol.

### Flow cytometry detection of ROS

ROS levels inside the cells were assessed using the DCFH-DA fluorescent probe (Servicebio, G1706, China). After the designated treatments, cells were treated with DCFH-DA and incubated at 37 °C for 30 min. Following the staining, the cells were trypsinized and subjected to flow cytometry analysis using a CytoFLEX system (Beckman Coulter).

### Transmission electron microscopy

Spinal tissue or HT22 cells were initially fixed with 2% glutaraldehyde for 2 h, followed by postfixation in 1% citric acid. Subsequently, the cells were stained with uranyl acetate and dehydrated through a graded acetone series. The specimens were subsequently encased in epoxy resin and sliced into ultrathin sections measuring 70 to 90 nm in thickness. Sections were mounted onto copper grids, counterstained with lead citrate, and examined for mitochondrial ultrastructure using transmission electron microscopy.

### Lipid peroxidation analysis

Cells were plated into 12-well plates and allowed to adhere overnight, followed by treatment with specific concentrations of erastin for 24 h. The cells were then exposed to 5 μM BODIPY 581/591 C11 (Amgicam, 217075-36-0, China). After a 30-min incubation at 37 °C, the cells were rinsed with PBS, and fluorescence images were obtained using an inverted fluorescence microscope (Nikon, Ti2-U).

### Measurements of mitochondrial ROS

Mitochondrial superoxide levels were quantified using the MitoSOX fluorescent probe, following the manufacturer’s guidelines. HT22 cells were treated with 2 μM MitoSOX-Green (SparkJade, SJ-MD0286, China) at 37 °C for 20 min and then washed with PBS. Fluorescence intensity was subsequently analyzed using flow cytometry.

### ER and mitochondrial morphology analysis by confocal microscopy

For the analysis of ER and mitochondrial morphology, HT22 cells were plated at a concentration of 5 × 10^4^ cells per 15-mm glass-bottom dish (Corning, USA). Following treatment, cells were stained with MitoTracker Deep Red (Invitrogen, M22425) or ER-Tracker (Invitrogen, E12353) for 15 min at 37 °C. After staining, cells were rinsed with PBS and maintained in DMEM for imaging. Confocal images were acquired using a ×100 oil immersion objective on a Nikon CSU-W1 Spinning Disk Confocal microscope, analyzed according to a previously reported method [47].

### MitoPeDPP

Mitochondrial lipid peroxidation was evaluated by detecting the oxidation of MitoPeDPP, following the manufacturer’s instructions. MitoPeDPP is a cell-permeable, perylene-based fluorescent probe that selectively accumulates in mitochondria due to the presence of a triphenylphosphonium moiety. Mitochondrial localization of the MitoPeDPP signal was confirmed by fluorescence microscopy in cells incubated with the probe.

### RNA sequencing and bioinformatics analysis

In addition, publicly available SCI-related transcriptomic datasets (GSE45006 and GSE166009) were obtained from the Gene Expression Omnibus (GEO) database. Following normalization, differential gene expression analysis was performed with the limma package in R. Genes showing an adjusted *P* value <0.05 and a |log_2_ fold change| ≥1 were classified as differentially expressed.

### RNA extraction and RT-qPCR

RNA was isolated with TRIzol reagent (Invitrogen, 15596026CN, USA), following the manufacturer’s protocol. Synthesis of cDNA was performed with the ReverTra Ace qPCR RT Kit (Foregene, RT-01022, China). Quantitative real-time polymerase chain reaction (qRT-PCR) was conducted using the SYBR Green PCR Kit (Foregene, QP-01012, China). Gene expression was normalized to β-tubulin. Relative expression was determined with the 2^−ΔΔCt^ method. Primer sequences for amplification can be found in Tables S1 and S2.

### ChIP–qPCR

ChIP was performed using a commercially available ChIP assay kit (Beyotime, P2078, China) in accordance with the manufacturer’s protocol. Subsequent quantification of immunoprecipitated DNA was carried out by qPCR. The enrichment of target regions was calculated and expressed as a percentage of input DNA.

### Western blotting assay

To prepare the samples, lysis was performed on ice using radioimmunoprecipitation assay buffer (EpiZyme, GRF101, China) containing a protease inhibitor cocktail, along with 10% sodium dodecyl sulfate, 0.1% Triton X-100, 2 mM EDTA, 50 mM Tris–HCl (pH 7.4), 10% sodium deoxycholate, and 150 mM NaCl. The lysates were separated via sodium dodecyl sulfate–polyacrylamide gel electrophoresis and subsequently transferred to polyvinylidene fluoride membranes (Millipore, Billerica, MA, USA). Membranes were then incubated for 1 h at room temperature in 5% nonfat milk for blocking, followed by overnight incubation with primary antibodies MFN-2 (Proteintech, 12186-1-AP, China), β-tubulin (Proteintech, 10094-1-AP, China), GAPDH (Proteintech, 10494-1-AP, China), CHOP (Wanleibio, WL00880, China), PERK (Wanleibio, WL03378, China), FIS1 (Proteintech, 10956-1-AP, China), SCD1 (Abcam, ab19862, UK), LXRα (Wanleibio, WL05098, China), GPX4 (Proteintech, 30388-1-AP, China), COX2 (Proteintech, 27308-1-AP, China), TOMM20 (Abcam, ab186735, UK), EZH2 (Proteintech, 21800-1-AP, China), and calreticulin (Abcam, ab2907, UK) at 4 °C. Following washing, membranes were incubated for 1 h at room temperature with horseradish peroxidase-linked secondary antibodies—either anti-mouse immunoglobulin G (IgG) (1:5,000, Cell Signaling Technology) or anti-rabbit IgG (1:5,000, Sigma-Aldrich)—with gentle agitation. Immunoreactive bands were then detected using an enhanced chemiluminescence substrate (Merck KGaA, Darmstadt, Germany), and images were captured using the ChemiDoc MP Imaging System (Bio-Rad, CA, USA).

### Isolation of mitochondria from cells

Mitochondria were extracted using the Cell Mitochondria Isolation Kit (Solarbio, EX2610, China), according to the manufacturer’s guidelines. After cell collection, they were lysed in precooled buffer containing a protease inhibitor cocktail (MedChemExpress) and homogenized thoroughly. The lysate was first centrifuged at 1,000 × g for 5 min to eliminate cell debris, and the supernatant was then spun at 12,000 × g for 10 min to isolate the mitochondrial pellet. We washed the pellets with mito-wash buffer and resuspended them in storage buffer for further use.

### Quantification and statistical analysis

Data are presented as mean ± standard deviation. Statistical evaluations were performed using the GraphPad Prism software, with the specific tests outlined in the corresponding figure legends. Statistical significance was considered for *P* values less than 0.05. The following significance levels were used: *P < 0.05, **P < 0.01, and ***P < 0.001.

## Data Availability

The transcriptomic datasets analyzed in this study were retrieved from the NCBI GEO database: GSE166009 and GSE45006. All datasets are publicly available for noncommercial research use via the GEO official portal. The other data that support the findings of this study are available on request from the corresponding authors.

## References

[1] Courtine G, Sofroniew MV. Spinal cord repair: Advances in biology and technology. Nat Med. 2019;25(6):898–908. 31160817 10.1038/s41591-019-0475-6

[2] Kumar R, Lim J, Mekary RA, Rattani A, Dewan MC, Sharif SY, Osorio-Fonseca E, Park KB. Traumatic spinal injury: Global epidemiology and worldwide volume. World Neurosurg. 2018;113:e345–e363. 29454115 10.1016/j.wneu.2018.02.033

[3] Ding W, Hu S, Wang P, Kang H, Peng R, Dong Y, Li F. Spinal cord injury: The global incidence, prevalence, and disability from the Global Burden of Disease Study 2019. Spine. 2022;47(21):1532–1540. 35857624 10.1097/BRS.0000000000004417 PMC9554757

[4] Sun S, Shen J, Jiang J, Wang F, Min J. Targeting ferroptosis opens new avenues for the development of novel therapeutics. Signal Transduct Target Ther. 2023;8(1):372. 37735472 10.1038/s41392-023-01606-1 PMC10514338

[5] Chopra M, Kumar H. Navigating the complexities of spinal cord injury: An overview of pathology, treatment strategies and clinical trials. Drug Discov Today. 2025;30(6): Article 104387. 40436265 10.1016/j.drudis.2025.104387

[6] Xiong J, Ge X, Pan D, Zhu Y, Zhou Y, Gao Y, Wang H, Wang X, Gu Y, Ye W, et al. Metabolic reprogramming in astrocytes prevents neuronal death through a UCHL1/PFKFB3/H4K8la positive feedback loop. Cell Death Differ. 2025;32(7):1214–1230. 40016338 10.1038/s41418-025-01467-x PMC12284030

[7] Liu W, Zhu Y, Ye W, Xiong J, Wang H, Gao Y, Huang S, Zhang Y, Zhou X, Zhou X, et al. Redox regulation of TRIM28 facilitates neuronal ferroptosis by promoting SUMOylation and inhibiting OPTN-selective autophagic degradation of ACSL4. Cell Death Differ. 2025;32(6):1041–1057. 39875520 10.1038/s41418-025-01452-4 PMC12162872

[8] Fu C, Jin X, Ji K, Lan K, Mao X, Huang Z, Chen J, Zhao F, Li P, Hu X, et al. Macrophage-targeted *Mms6* mRNA-lipid nanoparticles promote locomotor functional recovery after traumatic spinal cord injury in mice. Sci Adv. 2025;11(13): Article eads2295. 40138430 10.1126/sciadv.ads2295 PMC11939073

[9] Sassano ML, Tyurina YY, Diokmetzidou A, Vervoort E, Tyurin VA, More S, La Rovere R, Giordano F, Bultynck G, Pavie B, et al. Endoplasmic reticulum–mitochondria contacts are prime hotspots of phospholipid peroxidation driving ferroptosis. Nat Cell Biol. 2025;27(6):902–917. 40514428 10.1038/s41556-025-01668-z PMC12173944

[10] Zhao Y, Liu B, Xu L, Yu S, Fu J, Wang J, Yan X, Su J. ROS-induced mtDNA release: The emerging messenger for communication between neurons and innate immune cells during neurodegenerative disorder progression. Antioxidants. 2021;10(12):1917. 34943020 10.3390/antiox10121917 PMC8750316

[11] Wang Y, Wang M, Liu Y, Tao H, Banerjee S, Srinivasan S, Nemeth E, Czaja MJ, He P. Integrated regulation of stress responses, autophagy and survival by altered intracellular iron stores. Redox Biol. 2022;55: Article 102407. 35853304 10.1016/j.redox.2022.102407 PMC9294649

[12] Lorito N, Subbiani A, Smiriglia A, Bacci M, Bonechi F, Tronci L, Romano E, Corrado A, Longo DL, Iozzo M, et al. FADS1/2 control lipid metabolism and ferroptosis susceptibility in triple-negative breast cancer. EMBO Mol Med. 2024;16(7):1533–1559. 38926633 10.1038/s44321-024-00090-6 PMC11251055

[13] Mancardi D, Mezzanotte M, Arrigo E, Barinotti A, Roetto A. Iron overload, oxidative stress, and ferroptosis in the failing heart and liver. Antioxidants. 2021;10(12):1864. 34942967 10.3390/antiox10121864 PMC8698778

[14] Tracey TJ, Steyn FJ, Wolvetang EJ, Ngo ST. Neuronal lipid metabolism: Multiple pathways driving functional outcomes in health and disease. Front Mol Neurosci. 2018;11:10. 29410613 10.3389/fnmol.2018.00010 PMC5787076

[15] Huang J, Zhang C, Huang C, Deng K, Xiao Y, Gao W, Wu M, Lei M. Mitochondria metabolism regulates glucose–lipid homeostasis in neurodegenerative diseases. Research. 2025;8: Article 0912. 41049611 10.34133/research.0912 PMC12489184

[16] Huang W, Zhang Y, Das NK, Solanki S, Jain C, El-Derany MO, Koo I, Bell HN, Aabed N, Singhal R, et al. Fibroblast lipid metabolism through ACSL4 regulates epithelial sensitivity to ferroptosis in IBD. Nat Metab. 2025;7(7):1358–1374. 40571769 10.1038/s42255-025-01313-x PMC12444769

[17] Xie X, Tian L, Zhao Y, Liu F, Dai S, Gu X, Ye Y, Zhou L, Liu X, Sun Y, et al. BACH1-induced ferroptosis drives lymphatic metastasis by repressing the biosynthesis of monounsaturated fatty acids. Cell Death Dis. 2023;14:48. 36670112 10.1038/s41419-023-05571-z PMC9860034

[18] Sen U, Coleman C, Sen T. Stearoyl coenzyme A desaturase-1: Multitasker in cancer, metabolism, and ferroptosis. Trends Cancer. 2023;9(6):480–489. 37029018 10.1016/j.trecan.2023.03.003

[19] Liu Y, Wang Y, Lin Z, Kang R, Tang D, Liu J. SLC25A22 as a key mitochondrial transporter against ferroptosis by producing glutathione and monounsaturated fatty acids. Antioxid Redox Signal. 2023;39(1–3):166–185. 37051693 10.1089/ars.2022.0203 PMC10620438

[20] Tesfay L, Paul BT, Konstorum A, Deng Z, Cox AO, Lee J, Furdui CM, Hegde P, Torti FM, Torti SV. Stearoyl-CoA desaturase 1 protects ovarian cancer cells from ferroptotic cell death. Cancer Res. 2019;79(20):5355–5366. 31270077 10.1158/0008-5472.CAN-19-0369 PMC6801059

[21] Kagan VE, Mao G, Qu F, Angeli JPF, Doll S, Croix CS, Dar HH, Liu B, Tyurin VA, Ritov VB, et al. Oxidized arachidonic and adrenic PEs navigate cells to ferroptosis. Nat Chem Biol. 2017;13(1):81–90. 27842066 10.1038/nchembio.2238 PMC5506843

[22] Shiiba I, Ito N, Oshio H, Ishikawa Y, Nagao T, Shimura H, Oh K-W, Takasaki E, Yamaguchi F, Konagaya R, et al. ER-mitochondria contacts mediate lipid radical transfer via RMDN3/PTPIP51 phosphorylation to reduce mitochondrial oxidative stress. Nat Commun. 2025;16:1508. 39929810 10.1038/s41467-025-56666-4 PMC11811300

[23] Ganji R, Paulo JA, Xi Y, Kline I, Zhu J, Clemen CS, Weihl CC, Purdy JG, Gygi SP, Raman M. The p97-UBXD8 complex regulates ER-Mitochondria contact sites by altering membrane lipid saturation and composition. Nat Commun. 2023;14:638. 36746962 10.1038/s41467-023-36298-2 PMC9902492

[24] Penvose A, Keenan JL, Bray D, Ramlall V, Siggers T. Comprehensive study of nuclear receptor DNA binding provides a revised framework for understanding receptor specificity. Nat Commun. 2019;10:2514. 31175293 10.1038/s41467-019-10264-3 PMC6555819

[25] Chen L, Liu H, Jiang L, Wang Z, Chang Y, Li N, Feng S. Lipid droplets metabolism mediated by ANXA7-PPARγ signaling axis regulates spinal cord injury repair in mice. Adv Sci. 2025;12(16): Article e2417326. 10.1002/advs.202417326 PMC12021101 39996504

[26] Li H, Zhang X, Qi X, Zhu X, Cheng L. Icariin inhibits endoplasmic reticulum stress-induced neuronal apoptosis after spinal cord injury through modulating the PI3K/AKT signaling pathway. Int J Biol Sci. 2019;15(2):277–286. 30745820 10.7150/ijbs.30348 PMC6367543

[27] Hu X, Zhang H, Zhang Q, Yao X, Ni W, Zhou K. Emerging role of STING signalling in CNS injury: Inflammation, autophagy, necroptosis, ferroptosis and pyroptosis. J Neuroinflammation. 2022;19(1):242. 36195926 10.1186/s12974-022-02602-y PMC9531511

[28] Dixon SJ, Olzmann JA. The cell biology of ferroptosis. Nat Rev Mol Cell Biol. 2024;25:424–442. 38366038 10.1038/s41580-024-00703-5 PMC12187608

[29] Ntambi JM, Miyazaki M. Regulation of stearoyl-CoA desaturases and role in metabolism. Prog Lipid Res. 2004;43(2):91–104. 14654089 10.1016/s0163-7827(03)00039-0

[30] Yao S, Pang M, Wang Y, Wang X, Lin Y, Lv Y, Xie Z, Hou J, Du C, Qiu Y, et al. Mesenchymal stem cell attenuates spinal cord injury by inhibiting mitochondrial quality control-associated neuronal ferroptosis. Redox Biol. 2023;67: Article 102871. 37699320 10.1016/j.redox.2023.102871 PMC10506061

[31] Wozny MR, Di Luca A, Morado DR, Picco A, Khaddaj R, Campomanes P, Ivanović L, Hoffmann PC, Miller EA, Vanni S, et al. In situ architecture of the ER–mitochondria encounter structure. Nature. 2023;618(7963):188–192. 37165187 10.1038/s41586-023-06050-3 PMC7614606

[32] Li Y, Huang D, Jia L, Shangguan F, Gong S, Lan L, Song Z, Xu J, Yan C, Chen T, et al. LonP1 links mitochondria–ER interaction to regulate heart function. Research. 2023;6: Article 0175. 37333972 10.34133/research.0175 PMC10275618

[33] Naón D, Hernández-Alvarez MI, Shinjo S, Wieczor M, Ivanova S, Martins de Brito O, Quintana A, Hidalgo J, Palacín M, Aparicio P, et al. Splice variants of mitofusin 2 shape the endoplasmic reticulum and tether it to mitochondria. Science. 2023;380(6651): Article eadh9351. 37347868 10.1126/science.adh9351

[34] He Q, Pu J, Yuan A, Lau WB, Gao E, Koch WJ, Ma X-L, He B. Activation of liver-X-receptor α but not liver-X-receptor β protects against myocardial ischemia/reperfusion injury. Circ Heart Fail. 2014;7(6):1032–1041. 25277999 10.1161/CIRCHEARTFAILURE.114.001260 PMC4527689

[35] Xuan Y, Wang H, Yung MM, Chen F, Chan WS, Chan YS, Tsui SK, Ngan HY, Chan KK, Chan DW. SCD1/FADS2 fatty acid desaturases equipoise lipid metabolic activity and redox-driven ferroptosis in ascites-derived ovarian cancer cells. Theranostics. 2022;12(7):3534–3552. 35547771 10.7150/thno.70194 PMC9065188

[36] Ding Z, Pan Y, Shang T, Jiang T, Lin Y, Yang C, Pang S, Cui X, Wang Y, Feng XF, et al. URI alleviates tyrosine kinase inhibitors-induced ferroptosis by reprogramming lipid metabolism in p53 wild-type liver cancers. Nat Commun. 2023;14(1):6269. 37805657 10.1038/s41467-023-41852-z PMC10560259

[37] Yang G, Yang Y, Song Z, Chen L, Liu F, Li Y, Jiang S, Xue S, Pei J, Wu Y, et al. Spliceosomal GTPase Eftud2 deficiency-triggered ferroptosis leads to Purkinje cell degeneration. Neuron. 2024;112(20):3452–3469.e9. 39153477 10.1016/j.neuron.2024.07.020

[38] Hong C, Tontonoz P. Liver X receptors in lipid metabolism: Opportunities for drug discovery. Nat Rev Drug Discov. 2014;13(6):433–444. 24833295 10.1038/nrd4280

[39] Zhang Y, Breevoort SR, Angdisen J, Fu M, Schmidt DR, Holmstrom SR, Kliewer SA, Mangelsdorf DJ, Schulman IG. Liver LXRα expression is crucial for whole body cholesterol homeostasis and reverse cholesterol transport in mice. J Clin Invest. 2012;122(5):1688–1699. 22484817 10.1172/JCI59817 PMC3336978

[40] Zhang R, Wuerch E, Yong VW, Xue M. LXR agonism for CNS diseases: Promises and challenges. J Neuroinflammation. 2024;21(1):97. 38627787 10.1186/s12974-024-03056-0 PMC11022383

[41] Peng D, Hiipakka RA, Reardon CA, Getz GS, Liao S. Differential anti-atherosclerotic effects in the innominate artery and aortic sinus by the liver X receptor agonist T0901317. Atherosclerosis. 2009;203(1):59–66. 18639878 10.1016/j.atherosclerosis.2008.05.058 PMC2663528

[42] Li W, Gui Y, Guo C, Huang Y, Liu Y, Yu X, Zhang H, Wang J, Liu R, Mahaman YAR, et al. Molecular mechanisms of mitochondrial quality control. Transl Neurodegener. 2025;14(1):45. 40887660 10.1186/s40035-025-00505-5 PMC12400733

[43] Dimmer KS, Rapaport D. Mitochondrial contact sites as platforms for phospholipid exchange. Biochim Biophys Acta Mol Cell Biol Lipids. 2017;1862(1):69–80. 27477677 10.1016/j.bbalip.2016.07.010

[44] Kirchgessner TG, Sleph P, Ostrowski J, Lupisella J, Ryan CS, Liu X, Fernando G, Grimm D, Shipkova P, Zhang R, et al. Beneficial and adverse effects of an LXR agonist on human lipid and lipoprotein metabolism and circulating neutrophils. Cell Metab. 2016;24(2):223–233. 27508871 10.1016/j.cmet.2016.07.016

[45] Musheshe N, Oun A, Sabogal-Guáqueta AM, Trombetta-Lima M, Mitchel SC, Adzemovic A, Speek O, Morra F, van der Veen CHJT, Lezoualc’h F, et al. Pharmacological inhibition of Epac1 averts ferroptosis cell death by preserving mitochondrial integrity. Antioxidants. 2022;11(2):314. 35204198 10.3390/antiox11020314 PMC8868285

[46] Yuan M, Wang F, Sun T, Bian X, Zhang Y, Guo C, Yu L, Yao Z. Vitamin B_6_ alleviates chronic sleep deprivation-induced hippocampal ferroptosis through CBS/GSH/GPX4 pathway. Biomed Pharmacother. 2024;174: Article 116547. 38599059 10.1016/j.biopha.2024.116547

[47] Bolte S, Cordelières FP. A guided tour into subcellular colocalization analysis in light microscopy. J Microsc. 2006;224(3):213–232. 17210054 10.1111/j.1365-2818.2006.01706.x

