# Supplementary material for: LXRα/SCD1-Mediated Endoplasmic Reticulum-Mitochondria Crosstalk in Inhibiting Neuronal Ferroptosis after Spinal Cord Injury
Source: Research (Wash D C). 2026 Feb 10;9:1077. doi: 10.34133/research.1077 (PMC13274629; doi:10.34133/research.1077)
Supplement: Supplementary 1 — Figs. S1 to S6 [file research.1077.f1.zip › supplementary_materials.docx]

# **Supplementary Materials for**

## **LXR-α/SCD1-mediated Endoplasmic Reticulum-Mitochondria Crosstalk in Inhibiting Neuronal Ferroptosis after Spinal Cord Injury**

Pan Jiang *et al*.

*Corresponding author. Liu Bin Email: liubin6@mail.sysu.edu.cn

## **This PDF file includes:**

Supplementary Text

Figs. S1 to S6

Tables S1 to S2


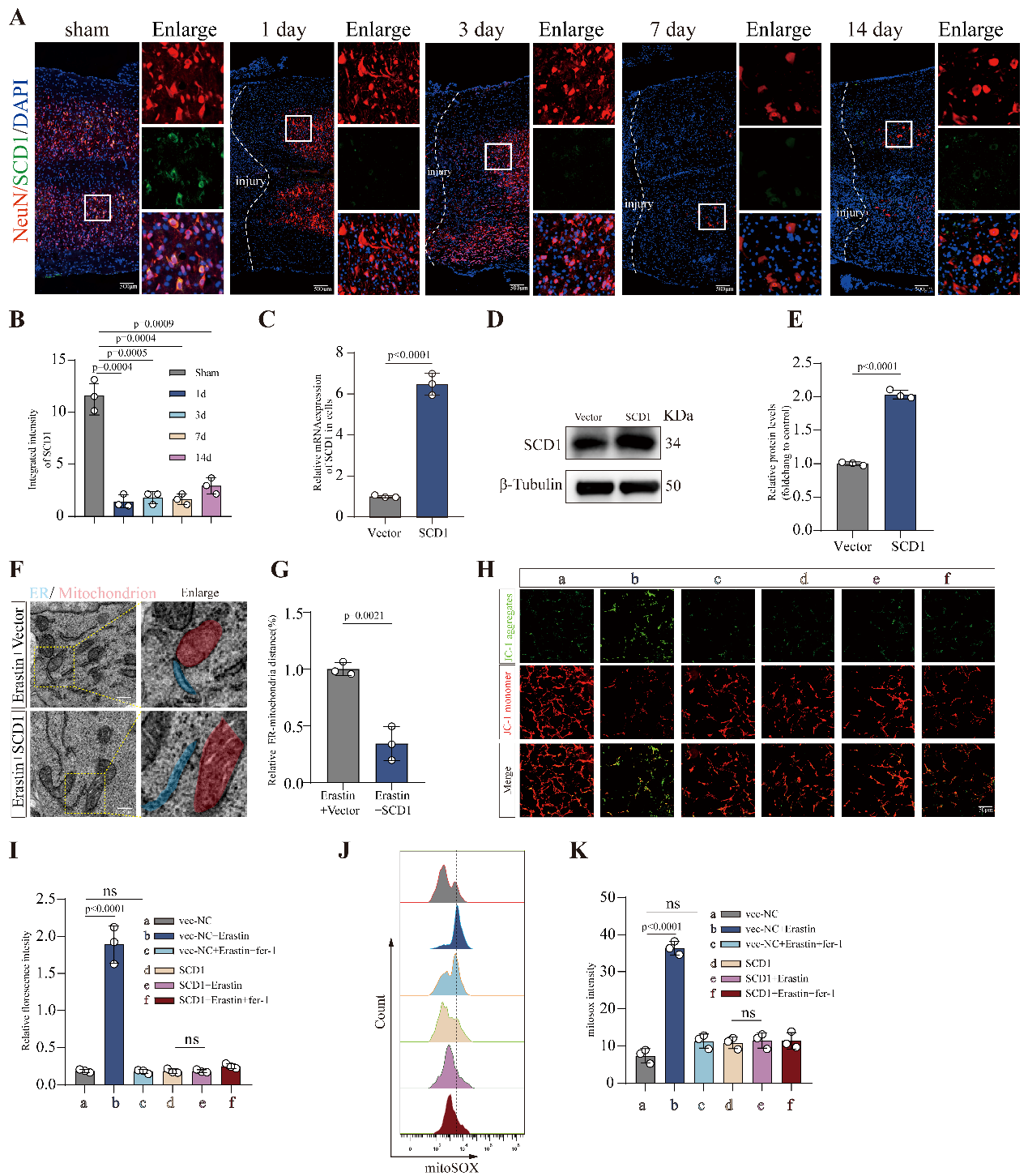


### ***Fig S1. Transcriptomic and functional validation of SCD1 in ferroptosis regulation.***

(A-B) Analysis of immunofluorescence revealed SCD1 downregulation at different time periods during SCI.

(C-E) qRT-PCR and western blot confirmed SCD1 overexpression in transfected HT22 cells.

(F-G) TEM analysis showed altered ER-mitochondria distance in erastin-treated cells, rescued by SCD1 overexpression.

(H-K) JC-1 and MitoSOX assays demonstrated restoration of mitochondrial membrane potential and reduction of ROS in SCD1-overexpressing cells. Data are mean ± SD (n = 3).


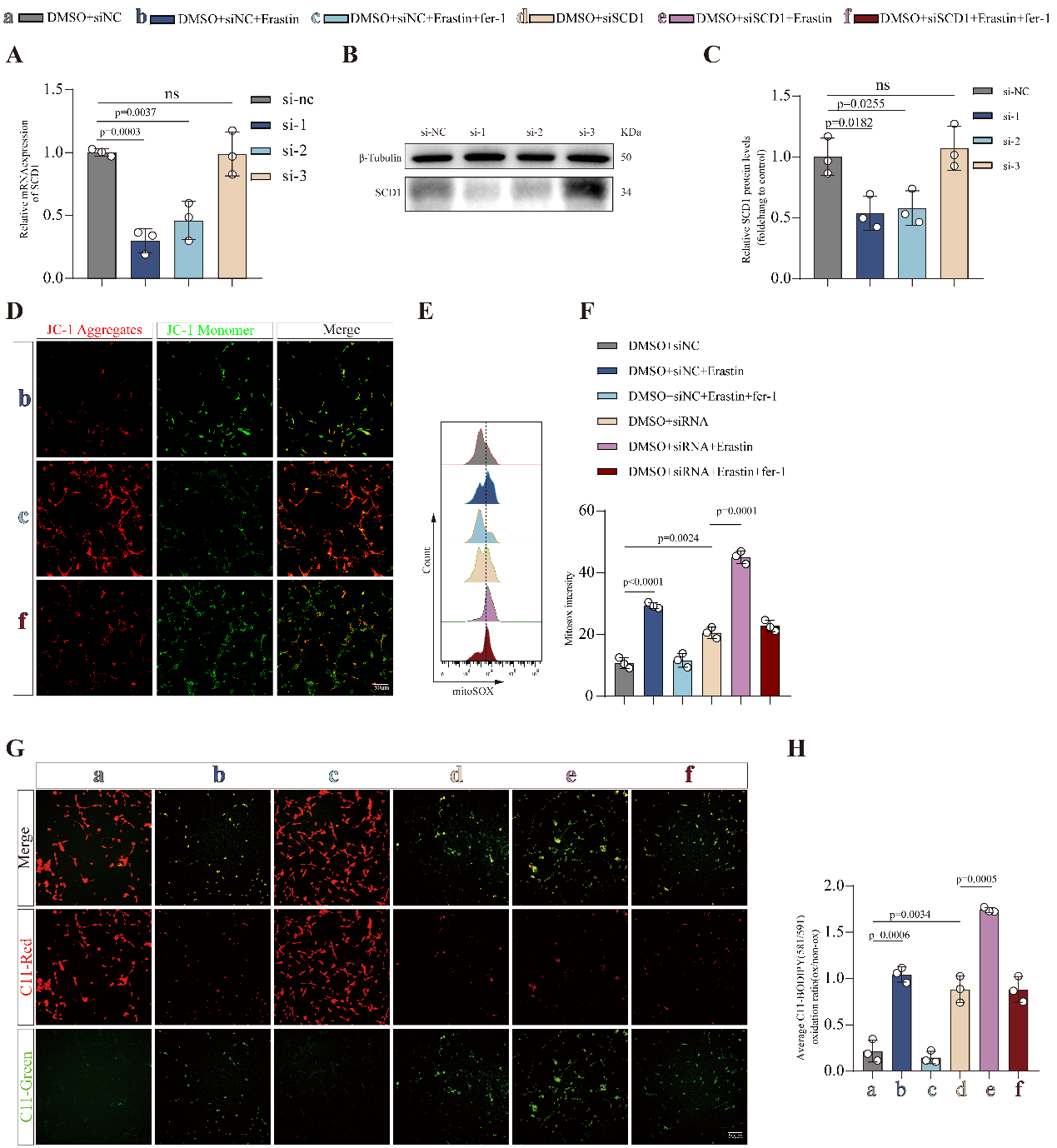


### ***Fig S2. Validation of SCD1 knockdown efficiency and functional outcomes.***

(A-C) qRT-PCR and western blot confirmed effective SCD1 knockdown by selected siRNAs.

(D-H) JC-1 staining, MitoSOX Red, and C11-BODIPY assays demonstrated mitochondrial depolarization, elevated ROS, and enhanced lipid peroxidation in SCD1-knockdown cells after erastin exposure, all mitigated by Fer-1. Data are mean ± SD (n = 3).


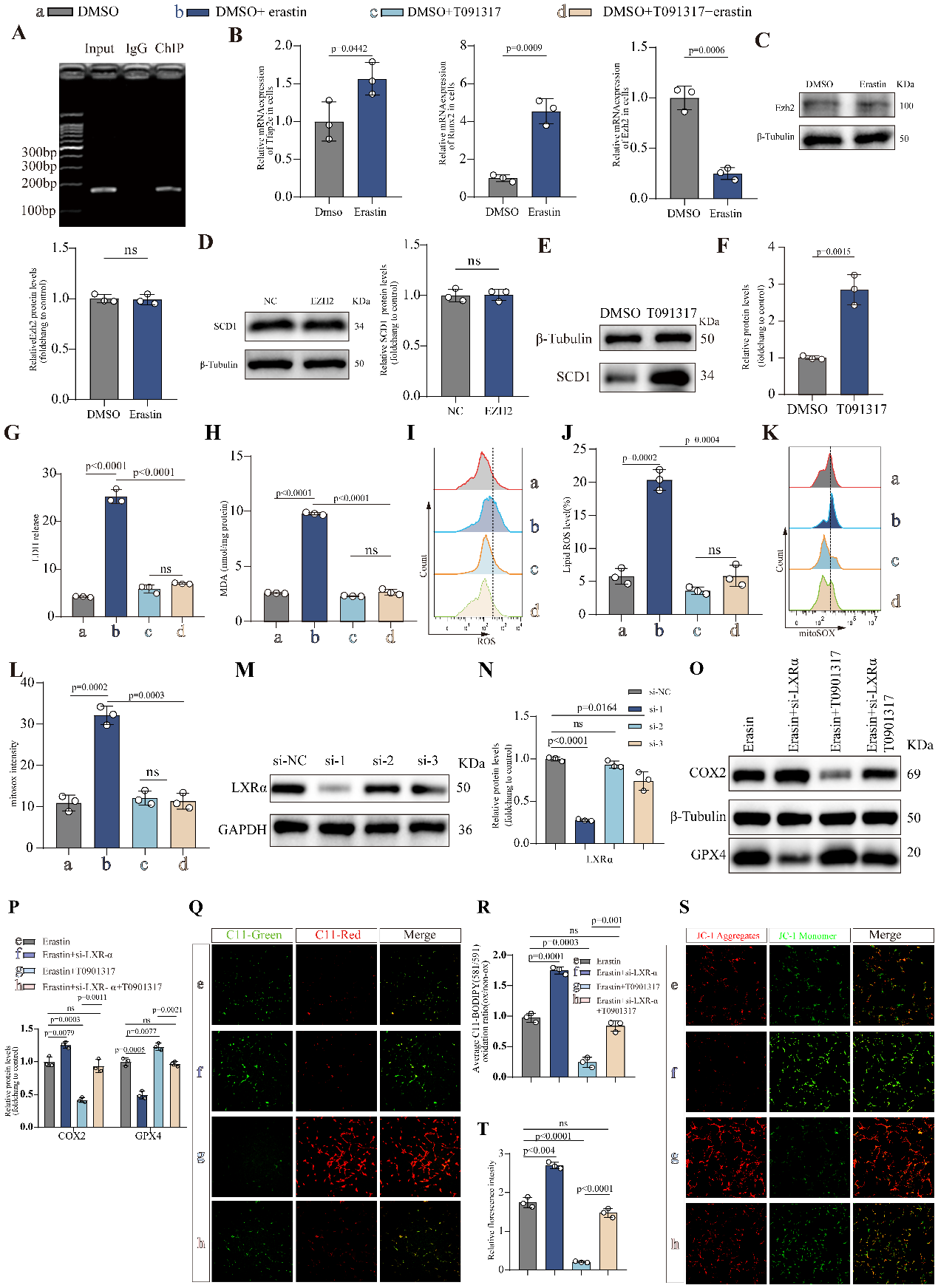


### ***Fig S3. LXRα acts as a transcription factor for SCD1 and plays a protective role against ferroptosis.***

(A) PCR analysis confirming the successful amplification of LXRα.

(B) qRT-PCR analysis of candidate transcription factors (Tfap2c, Runx2, and Ezh2) following erastin treatment.

(C) Western blot analysis of candidate transcription factor EZH2 expression after erastin induction.

(D) Western blot analysis showing expression of SCD1 following EZH2 overexpression.

(E, F) Western blot analysis demonstrating SCD1 upregulation after T0901317 treatment.

(G, H) LDH and MDA assays indicating reduced cellular damage and lipid peroxidation upon LXRα activation.

(I-L) T0901317 reduces erastin-induced reactive oxygen species (ROS) and mitochondrial superoxide.

(M-N) Representative Western blot image showing LXRα knockdown by siRNA.

(O-P) COX2 and GPX4 expression upon T0901317 treatment with or without LXRα knockdown by Western blot.

(Q-S) Functional assays (lipid peroxidation, JC-1) showing that T0901317 attenuated ferroptosis with or without LXRα knockdown. Data are mean ± SD (n = 3).


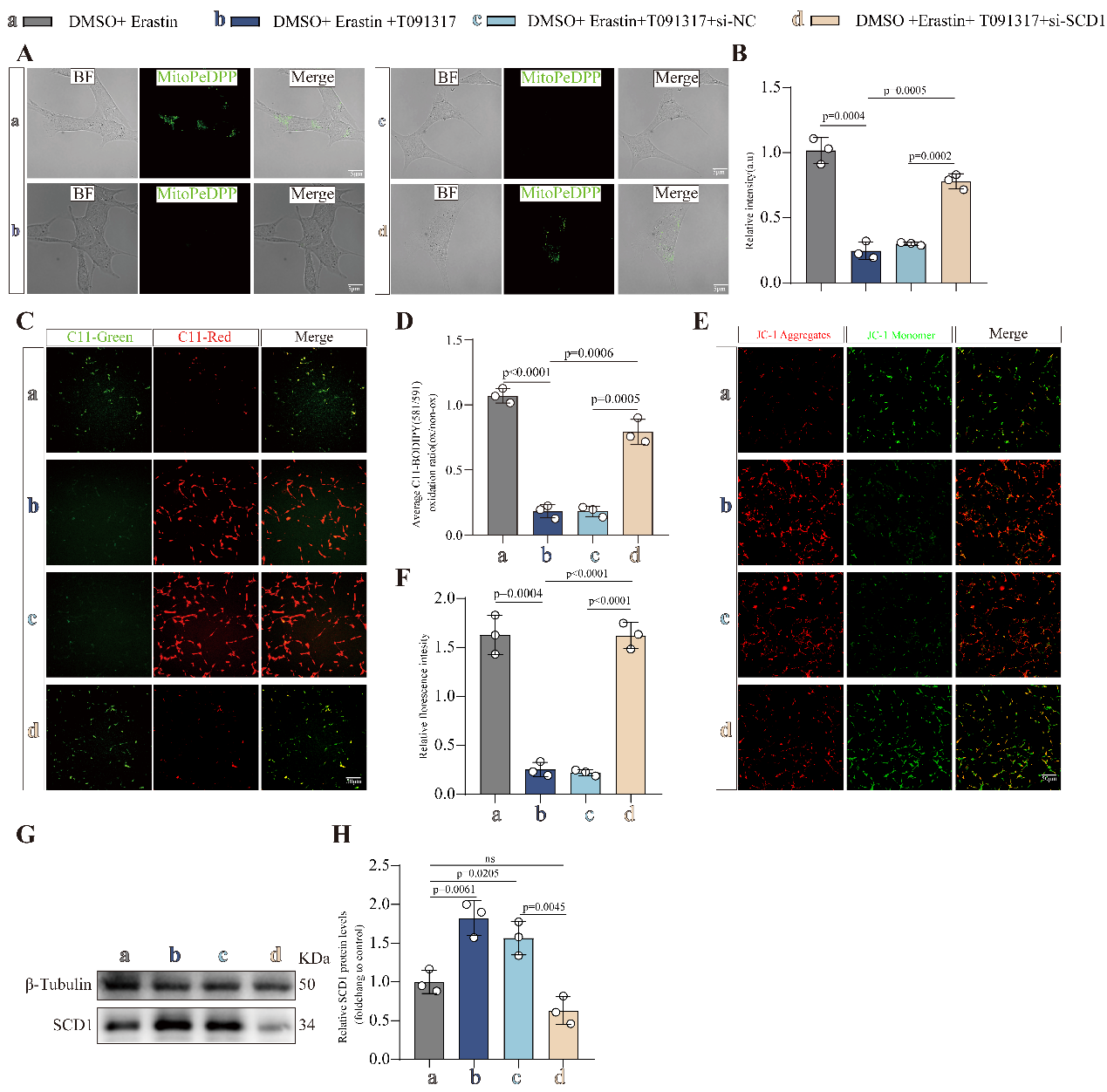


### ***Fig S4. Supplementary validation of the LXRα-SCD1 axis in regulating mitochondrial lipid peroxidation.***

(A-D) MitoPeDPP and C11-BODIPY staining demonstrated that T0901317 reduced mitochondrial and whole-cell lipid peroxidation in an SCD1-dependent manner.

(E-F) JC-1 staining confirmed preservation of mitochondrial membrane potential.

(G-H) Western blot validated efficient SCD1 knockdown in HT22 cells. Data are mean ± SD (n = 3).


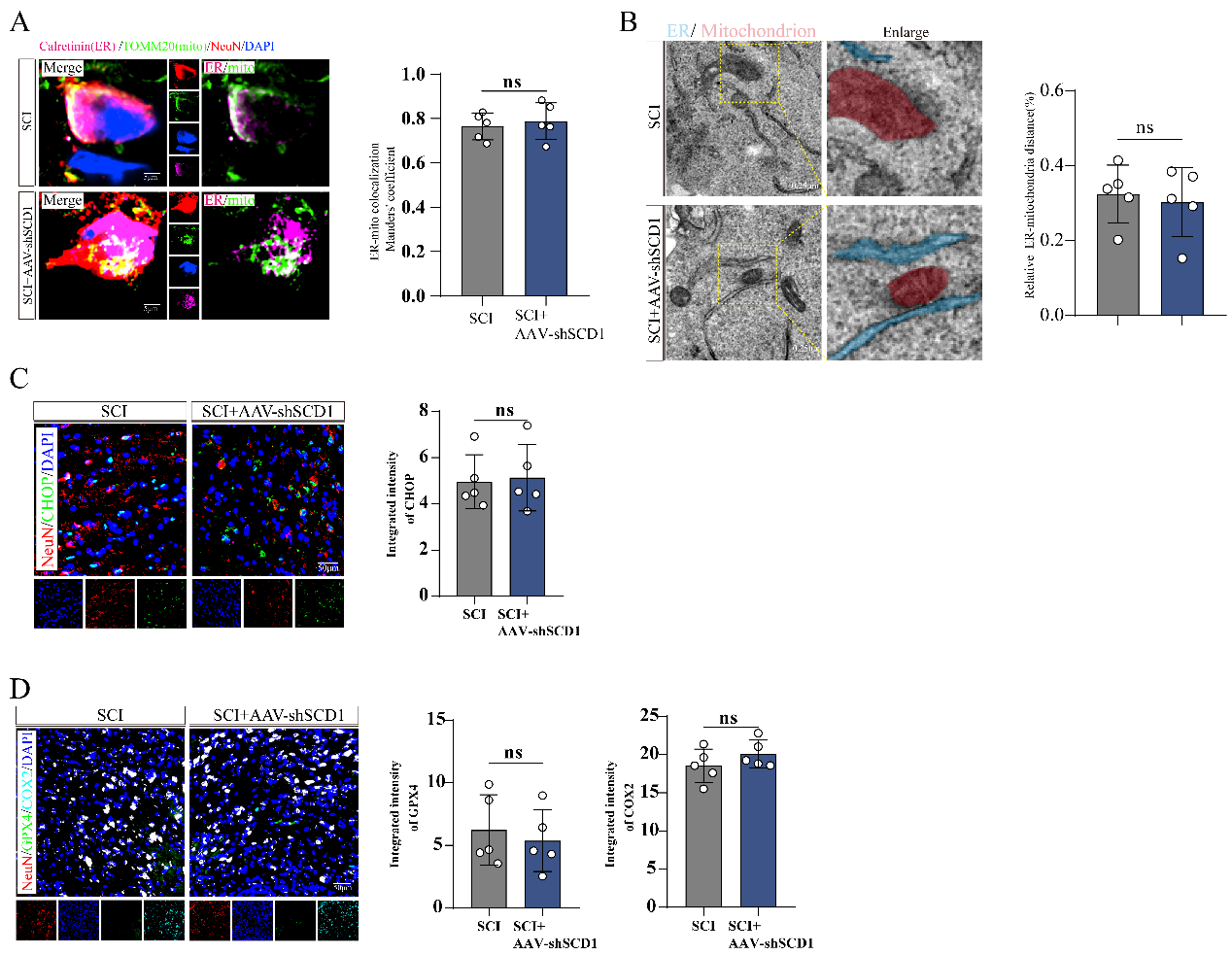


### ***Fig S5. SCD1 knockdown exacerbates post-SCI ER-mitochondria dysfunction.***

(A-B) Triple immunofluorescence and TEM analysis demonstrated reduced ER-mitochondria contacts, increased spacing, and cristae fragmentation in SCI+AAV-shSCD1 neurons compared with controls.

(C) Quantification showed increased CHOP+ neuron density in lesion border zones.

(D) Ferroptosis markers revealed exacerbated COX2/GPX4 imbalance in SCD1-deficient neurons.

Data are mean ± SD (n = 5). *p < 0.05, **p < 0.01, ***p<0.001.


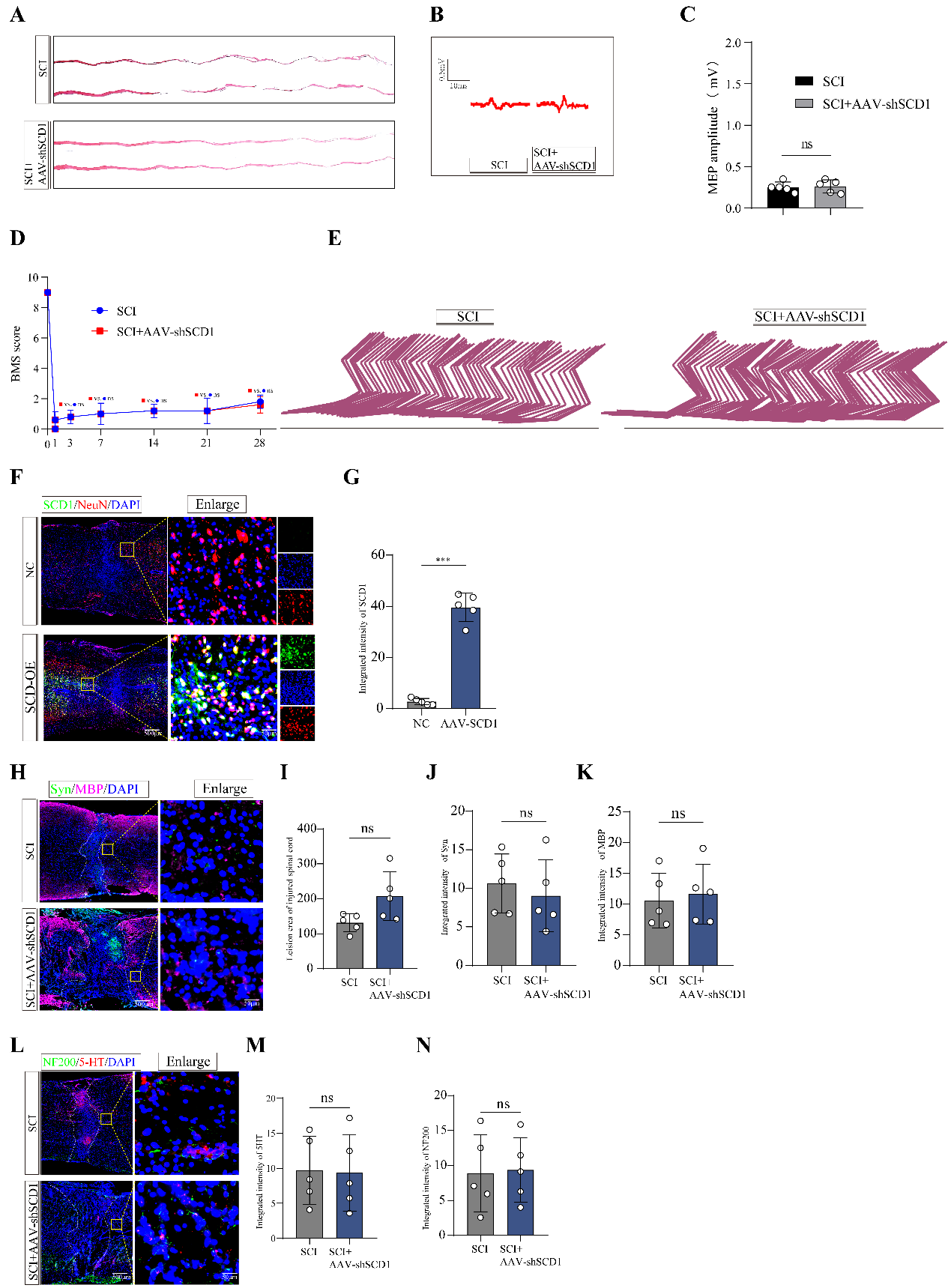


### ***Fig S6. SCD1 knockdown impairs long-term functional recovery.***

(A-E) Behavioral and electrophysiological assessments (footprints, sciatic nerve conduction, BMS scores, and gait kinematics) showed worsened outcomes in SCD1-deficient mice.

(F-G) Validation confirmed increased neuronal SCD1 expression at 1week post-SCI.

(H-K) Synaptic (Syn) and myelin (MBP) markers revealed reduced synaptic density and demyelination.

(L-N) Axonal pathology demonstrated loss of 5-HT+ fibers and NF200+ axons in SCD1-deficient spinal cords. Data are mean ± SD (n = 5). *p < 0.05, **p < 0.01, ***p<0.001.

### ***Table***

Table S1

Primer sequence

| gene | primer |
| --- | --- |
| SCD1 | Sense:TTCTTGCGATACACTCTGGTGC |
|  | Antisense: CGGGATTGAATGTTCTTGTCGT |
| ChIP-qPCR Primer | Sense: GGAAGCTCCTGAAGTCTACAGT |
|  | Antisense: CCTAGTCCAGGTAATATCCCCAG |

Table S2

Candidate transcription factors, sequences and descriptions

| Candidate transcription factor | Primer | Description |
| --- | --- | --- |
| fosl1 | Sense:ATGTACCGAGACTACGGGGAA | Low expression cannot be amplified |
|  | Antisense: CTGCTGCTGTCGATGCTTG |  |
| spi1 | Sense:CCAACGTCCAATGCATGACTA | Low expression cannot be amplified |
|  | Antisense: TGTGCGGAGAAATCCCAGTA |  |
| lmnb1 | Sense:CCGGCCTCAAGGCTCTCTA | Low expression cannot be amplified |
|  | Antisense: GTGCCGCCTCATACTCTCG |  |
| Fos | Sense：CGGGTTTCAACGCCGACTA | Low expression cannot be amplified |
|  | Antisense:TGGCACTAGAGACGGACAGAT |  |
| Irf5 | Sense：GCCACCTCAGCCGTACAAG | Low expression cannot be amplified |
|  | Antisense:CTCCCAGAACGTAATCATCAGTG |  |
| Cebpb | Sense：GCGGGAACGCAACAACATC | Low expression cannot be amplified |
|  | Antisense: GTCACTGGTCAACTCCAGCAC |  |
| Irf1 | Sense:ATGCCAATCACTCGAATGCG | Low expression cannot be amplified |
|  | Antisense:CCTGCTTTGTATCGGCCTGT |  |
| Tfap2c | Sense:TACCAGCCGCCTCCTTACTT | Upregulation. More data can be found in S3B |
|  | Antisense: TCCAGCCCTGAAATATGGGGT |  |
| Runx2 | Sense:TTCAACGATCTGAGATTTGTGGG | Upregulation. More data can be found in S3B |
|  | Antisense: GGATGAGGAATGCGCCCTA |  |
|  |  |  |
| Ezh2 | sense: AGCACAAGTCATCCCGTTAAAG | Downregulation, but can’t upregulate SCD1. More data can be found in Fig.S3B-C-D |
|  | Antisense：AATTCTGTTGTAAGGGCGACC |  |
| Nr1h3(LXRα) | sense: CTGATTCTGCAACGGAGTTGT | Downregulation More data can be found in Fig.4A-H |
|  | Antisense：GACGAAGCTCTGTCGGCTC |  |
