# Supplementary figures and images for: LXRα/SCD1-Mediated Endoplasmic Reticulum-Mitochondria Crosstalk in Inhibiting Neuronal Ferroptosis after Spinal Cord Injury
Source: Research (Wash D C). 2026 Feb 10;9:1077. doi: 10.34133/research.1077 (PMC13274629; doi:10.34133/research.1077)

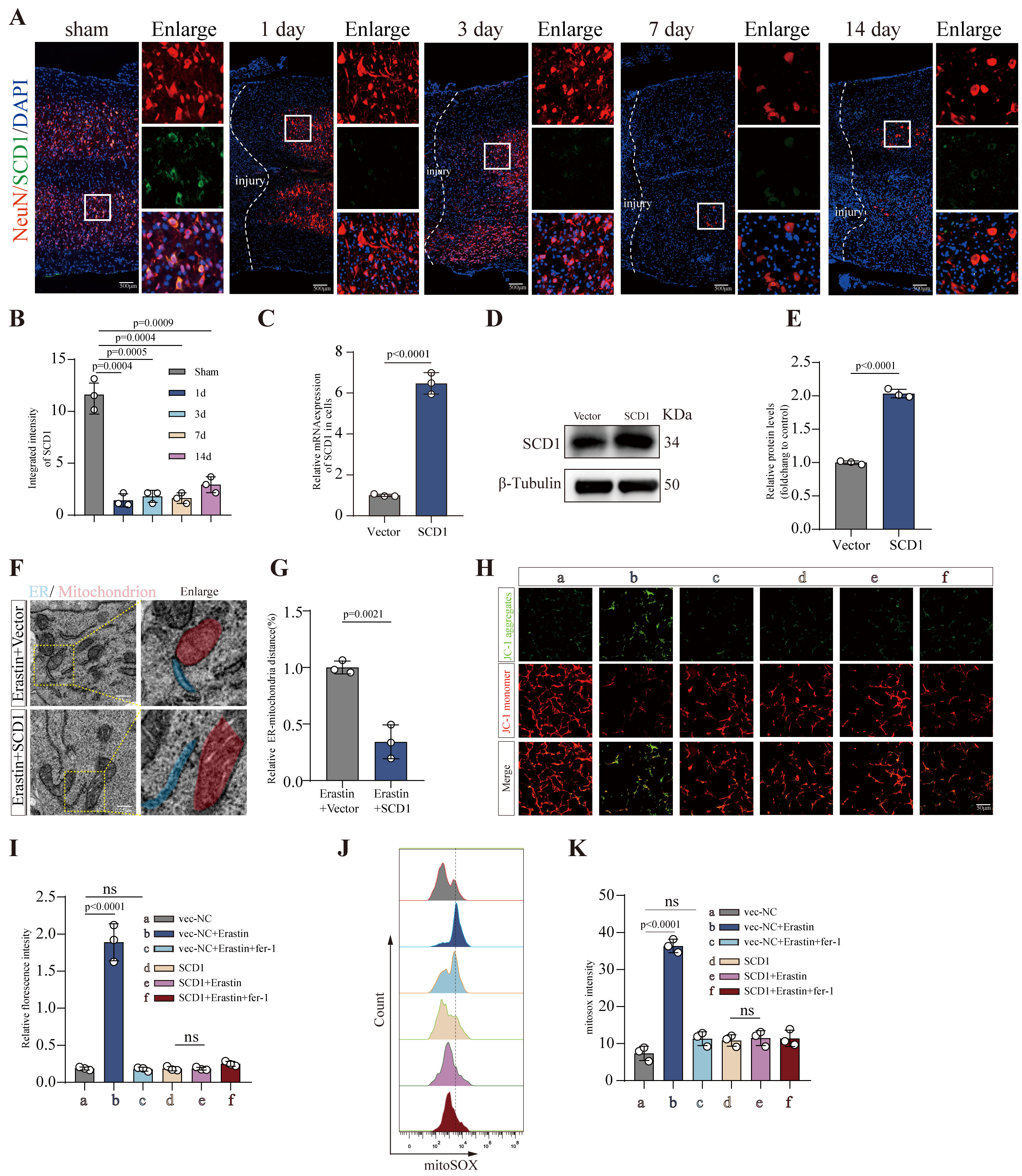

Supplement: Supplementary 1 — Figs. S1 to S6 [file research.1077.f1.zip › figureS1.tif]

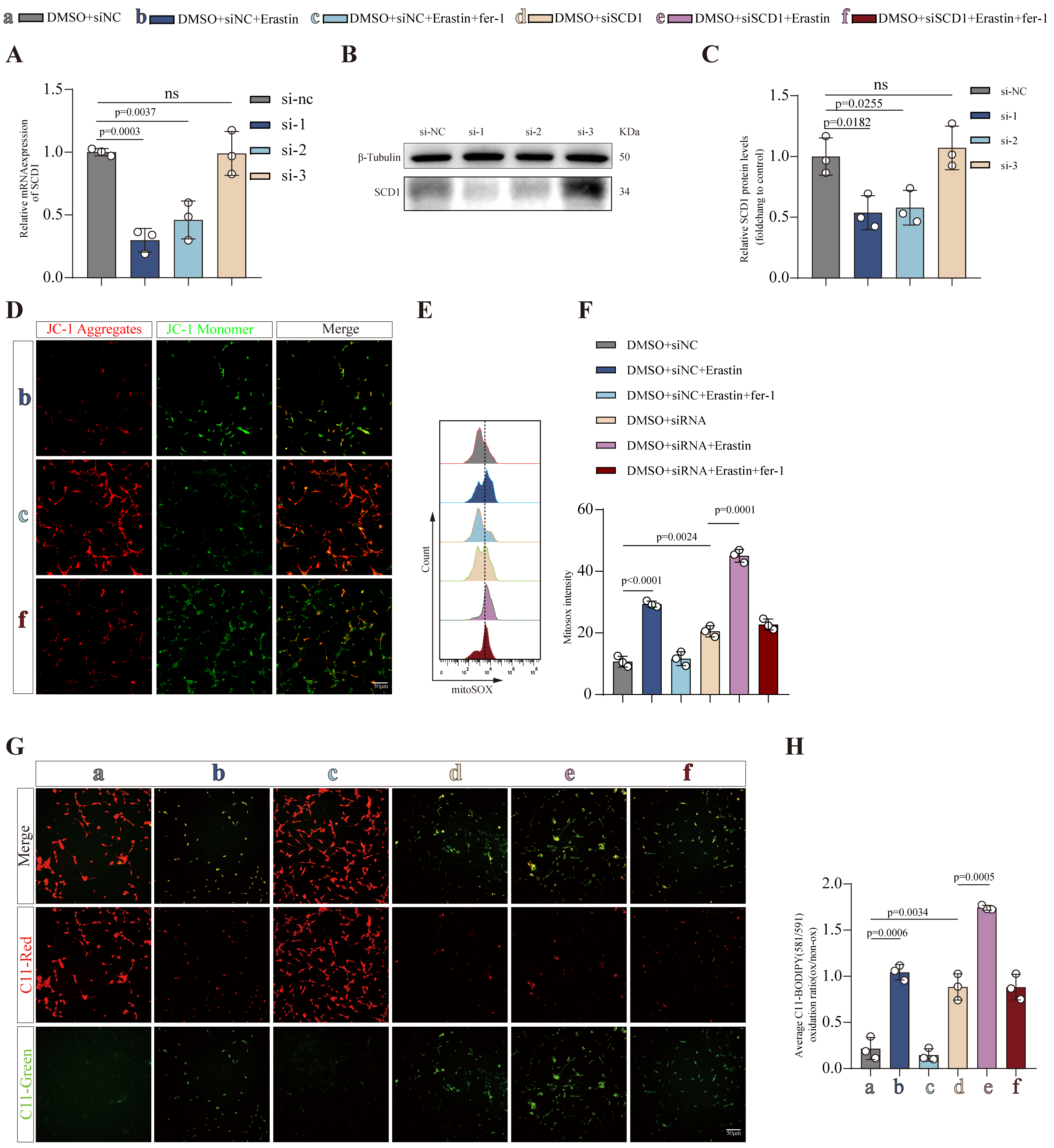

Supplement: Supplementary 1 — Figs. S1 to S6 [file research.1077.f1.zip › figureS2.tif]

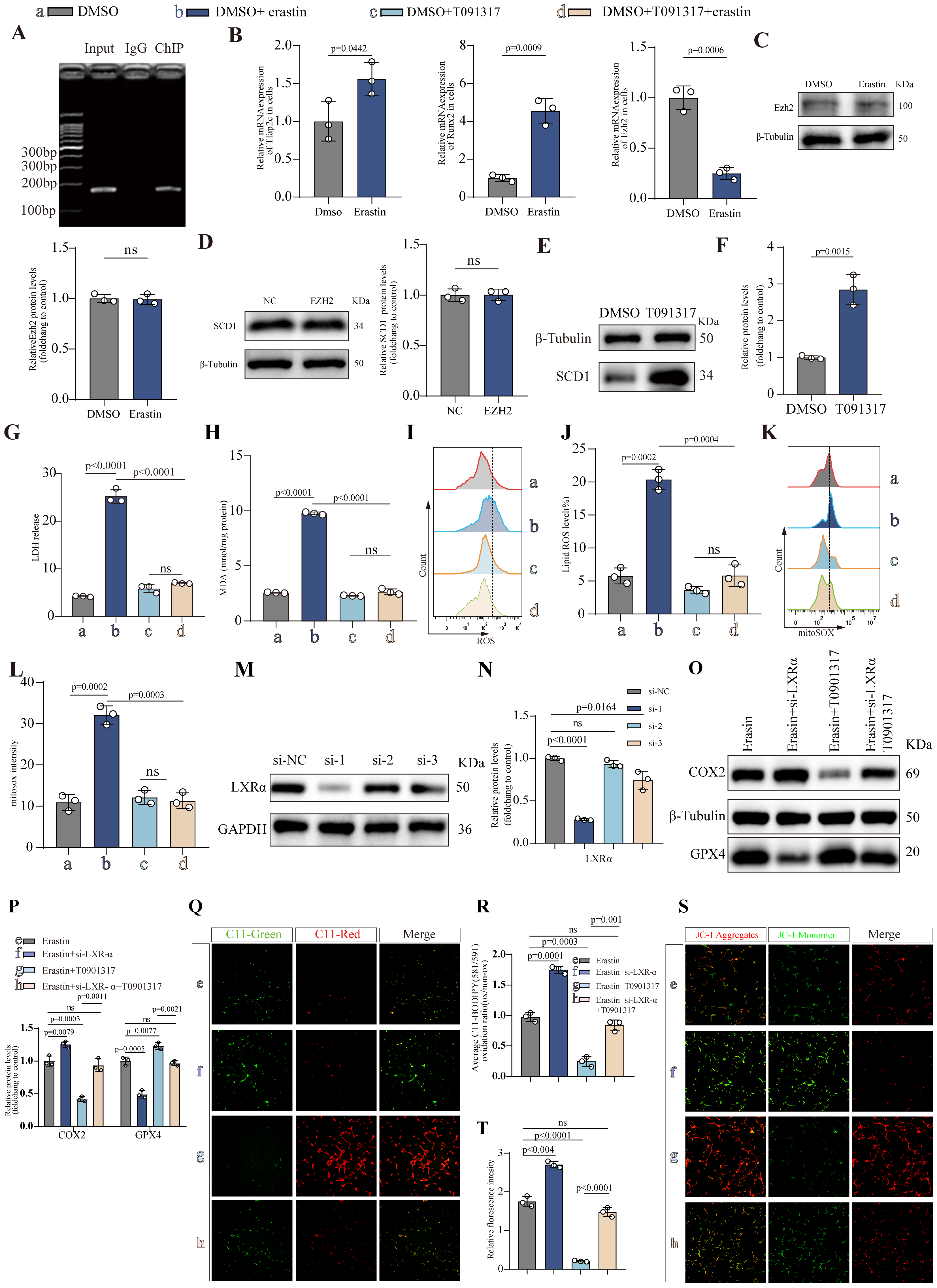

Supplement: Supplementary 1 — Figs. S1 to S6 [file research.1077.f1.zip › figureS3.tif]

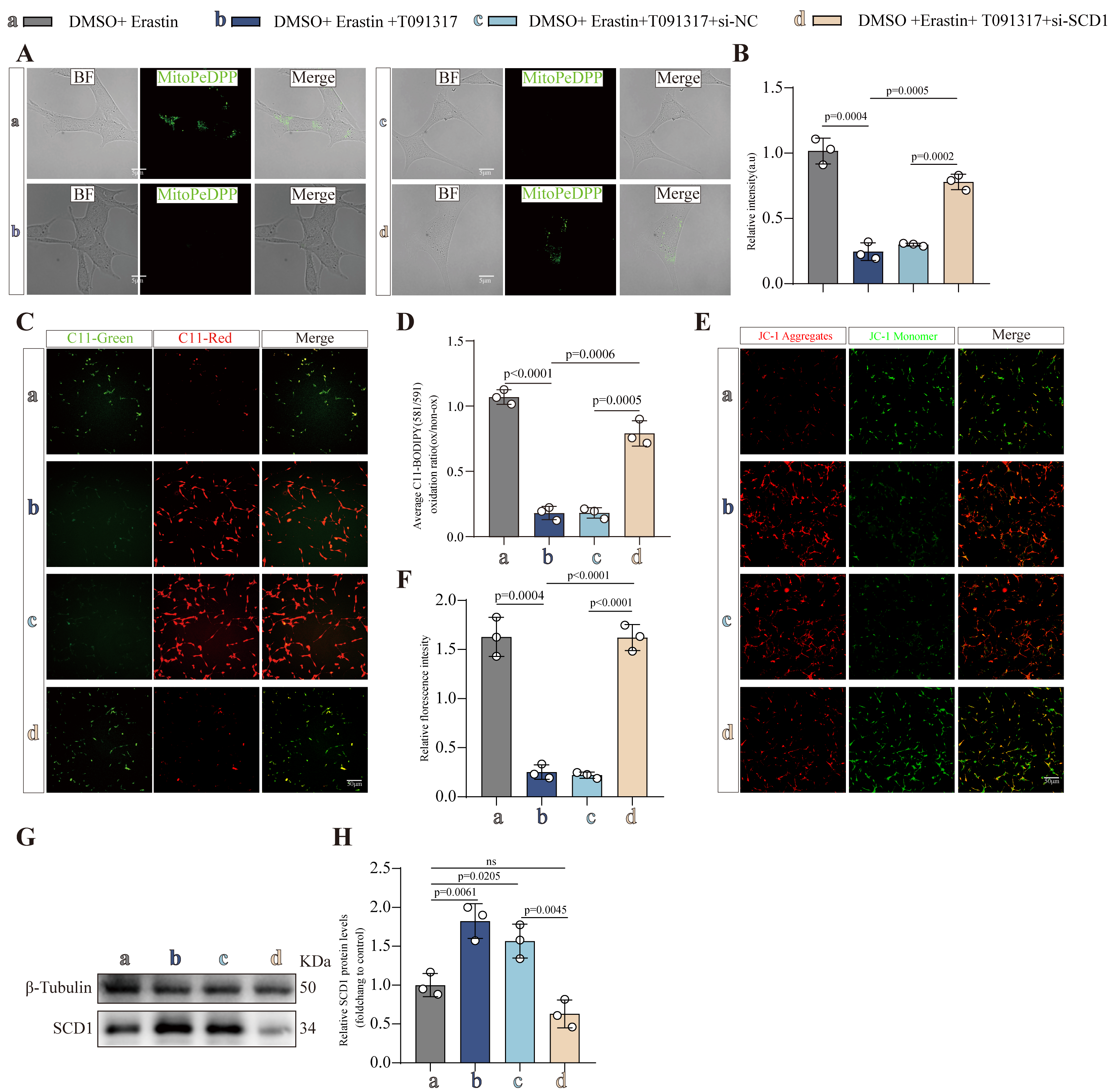

Supplement: Supplementary 1 — Figs. S1 to S6 [file research.1077.f1.zip › figureS4.tif]

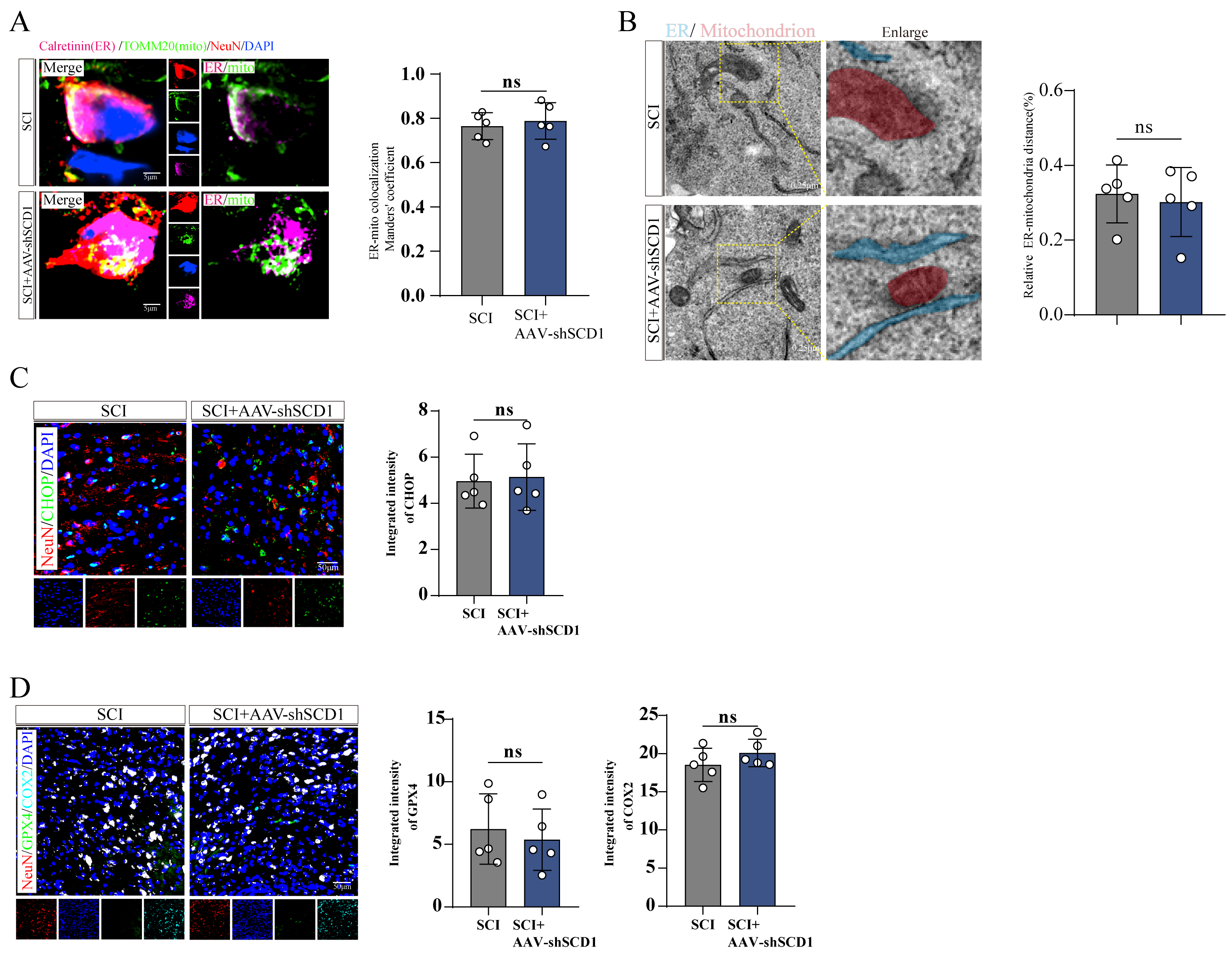

Supplement: Supplementary 1 — Figs. S1 to S6 [file research.1077.f1.zip › figureS5.tif]

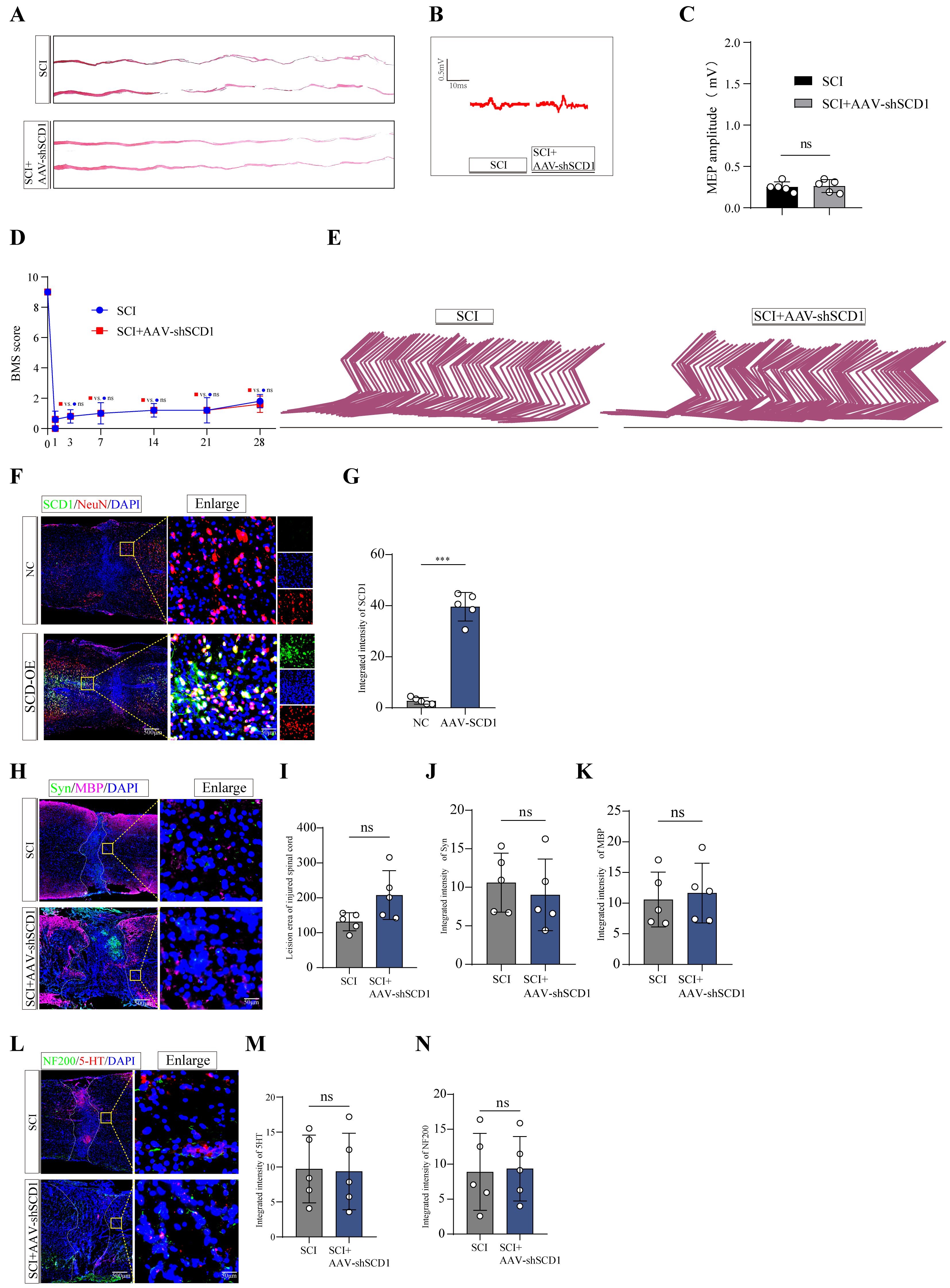

Supplement: Supplementary 1 — Figs. S1 to S6 [file research.1077.f1.zip › figureS6.tif]
